# Unraveling the spatial landscape of dystrophinopathies: a transcriptomic approach to Becker and Duchenne muscular dystrophies

**DOI:** 10.1002/path.70067

**Published:** 2026-05-01

**Authors:** Laura GM Heezen, Qirong Mao, Stefan Nicolau, Claudio Novella Rausell, Julia van der Weerd, Jan Kueckelhaus, Rasya Gokul Nath, Jordi Diaz‐Manera, Hermien E Kan, Erik H Niks, Maaike van Putten, Annemieke Aartsma‐Rus, Kevin M Flanigan, Ahmed Mahfouz, Pietro Spitali

**Affiliations:** ^1^ Department of Human Genetics Leiden University Medical Center Leiden The Netherlands; ^2^ Center for Gene Therapy The Abigail Wexner Research Institute at Nationwide Children's Hospital Columbus OH USA; ^3^ Microenvironment and Immunology Research Laboratory, Medical Center, Faculty of Medicine Freiburg University Freiburg Germany; ^4^ Department of Neurosurgery, Medical Center, Faculty of Medicine Erlangen University Erlangen Germany; ^5^ John Walton Muscular Dystrophy Research Centre Newcastle University Translational and Clinical Research Institute, Newcastle Upon Tyne NHS Trust Newcastle Upon Tyne UK; ^6^ Laboratori de Malalties Neuromusculars Institu de recerca Hospital Sant Pau Barcelona Spain; ^7^ Centro de Investigación Biomédica en red de enfermedades raras (CIBERER) Madrid Spain; ^8^ Department of Radiology Leiden University Medical Center Leiden The Netherlands; ^9^ Duchenne Center The Netherlands; ^10^ Department of Neurology Leiden University Medical Center Leiden The Netherlands; ^11^ Department of Pediatrics The Ohio State University Columbus OH USA; ^12^ Department of Neurology The Ohio State University Columbus OH USA; ^13^ Delft Bioinformatics Lab Delft University of Technology Delft The Netherlands

**Keywords:** spatial transcriptomics, dystrophinopathy, fibroadipogenic progenitors, histopathology, cell–cell communication, single‐nucleus RNA sequencing

## Abstract

Dystrophinopathies are caused by pathogenic variants in the *DMD* gene, resulting in partial (Becker) or complete loss (Duchenne) of dystrophin. Becker (BMD) and Duchenne muscular dystrophy (DMD) are characterized by progressive muscle wasting, fatty replacement, fibrosis, and loss of function. To study histopathological changes, we used Visium spatial transcriptomics to profile skeletal muscle biopsies of patients affected by dystrophinopathy (*n* = 8) and healthy controls (*n* = 4). We estimated the proportion of cell types and their spatial localization across samples applying a deconvolution strategy using previously published single‐nucleus RNA‐sequencing data. We identified genes enriched in fat patches and cell types such as fibroadipogenic progenitors (FAPs) in areas of active pathology. Using expression data of ligand–receptor pairs, we highlight cell–cell communications leading to fibrotic and adipogenic lesions. Finally, analysis of gene expression gradients in areas of adjacent muscle and fat, allowed the identification of genes associated with muscle areas committed to becoming fat. © 2026 The Author(s). *The Journal of Pathology* published by John Wiley & Sons Ltd on behalf of The Pathological Society of Great Britain and Ireland.

## Introduction

Becker muscular dystrophy (BMD) and Duchenne muscular dystrophy (DMD), collectively known as dystrophinopathies (DYSs), are caused by mutations in the *DMD* gene, encoding the dystrophin protein. BMD patients experience a milder disease progression compared to DMD patients as a consequence of pathogenic variants leading to reduced levels of partially functional dystrophin, while dystrophin is severely reduced or absent in DMD [1].

Absence of dystrophin leads to loss of muscle fiber stability as the protein acts as a linker between the intracellular F‐actin cytoskeleton and the extracellular matrix. As a consequence, muscle fibers are susceptible to contraction‐induced damage, which in turn leads to chronic inflammation, cycles of impaired regeneration and degeneration, and, finally, fibrofatty replacement of the muscle fibers [2].

Previous omics approaches, *in vitro* experiments, and histological studies uncovered gene expression signatures associated with muscle damage and the characteristic lesions typically observed in muscle biopsies of patients affected by dystrophinopathies. Such studies highlight the interplay of cell types required for successful regeneration in healthy skeletal muscles and how the events leading to successful regeneration are not synchronized in dystrophic muscles.

These studies showed that, upon acute damage, a subpopulation of muscle‐resident stem cells, fibroadipogenic progenitors (FAPs), proliferate and secrete trophic factors to stimulate myogenic activity of satellite cells. In a healthy situation, damaged fibers are regenerated with the help of satellite cells, whereas debris are removed by macrophages [3, 4, 5], and FAPs repair the connective tissue. The satellite cells proliferate and differentiate into myoblasts, which fuse into multinucleated myotubes and eventually lead to the formation of new myofibers [6]. However, the regenerative capacity of myofibers is impaired in patients suffering from a dystrophinopathy when the satellite cells become exhausted due to chronic injury [2, 7]. This, in turn, causes the FAPs to continue proliferating instead of ceasing their activity and differentiate into adipocytes or fibroblasts, resulting in increased depositions of fat and fibrotic lesions in the tissue [8, 9].

The mechanisms by which muscle tissue is replaced by fibrofatty tissue in dystrophinopathies, including the driving factors underlying genes and cell types, remain poorly understood. Understanding such processes is, however, essential to uncover the pathophysiology of the disease and to identify potential therapeutic targets as well as disease biomarkers. In this study, we retrospectively analyzed biopsies from eight patients with dystrophinopathy using spatial transcriptomics to obtain paired histological and gene expression data, with the aim of describing changes in cell types and gene expression in association with histological lesions.

## Materials and methods

### Ethical approval and patient consent

The Leiden University Medical Center (LUMC) biobank approved this research (3.4258/010/FB/jr), and informed consent forms were obtained from one DMD patient and his legal representatives. The healthy controls included in this study from the Netherlands were obtained in The Hague, from individuals undergoing anterior cruciate ligament (ACL) surgery. For these subjects (*n =* 3), the study was approved by the local Medical Ethical Review Board of The Hague Zuid‐West and the Erasmus Medical Centre and was conducted in accordance with the ethical standards stated in the 1964 Declaration of Helsinki and its later amendments (Algemeen formulier voor Beoordeling en Registratie number: NL54081.098.16). All other subjects (*n =* 1 healthy control, *n =* 7 DYS) were included in the USA and this study was approved by the Nationwide Children's Hospital Institutional Review Board (0502HSE046 and IRB14‐00719). All subjects or their guardians provided written informed consent prior to participation. Patients were included in their respective dystrophinopathy group based on clinical diagnosis.

Skeletal muscle biopsies were obtained for healthy controls in the Netherlands after ACL construction. These biopsies were taken by percutaneous biopsy (modified from [48]) using a minimally invasive biopsy needle. All biopsies were immediately frozen in liquid nitrogen and kept at −80 °C until further processing. For all biopsies included from the USA, patient muscle samples were collected from the quadriceps muscle, while the healthy control sample was collected from the hamstrings. All samples were frozen in isopentane chilled with liquid nitrogen, according to standard techniques.

For this study, we selected samples containing a combination of muscle fibers, adipose tissue, and fibrous connective tissue.

### Spatial Visium experiments

All biopsies (*n* = 12) were fresh frozen upon collection and cryosectioned in cross‐sections 10 μm thick at −22 °C using a CryoStar NX70 cryostat (Thermo Fisher Scientific, Waltham, MA, USA) before being placed on one of the five Visium Spatial Gene Expression slides (PN: 2000233, 10x Genomics) in either Leiden or Columbus (for a detailed overview, see supplementary material, Table S7). The cross‐sections were adhered to the slide by warming the back of the slides and were stored at −80 °C until further processing. The Visium Spatial Gene Expression slides were processed following the manufacturer's protocols. The slides were transferred on dry ice to the PCR machine with slide adaptor and warmed to 37 °C for 1 min prior to fixation in ice‐cold methanol for 30 min at −20 °C. Slides were then incubated with isopropanol at room temperature (RT) for 1 min, after which the H&E staining (Agilent Technologies, Santa Clara, CA, USA; Sigma‐Aldrich, Burlington, MA, USA) was performed following the 10x protocol. Images of the capture areas were taken using the ZEISS Axio Scan.Z1 slide Scanner (Carl Zeiss LTD, CAM, UK) with a 20× objective in Leiden and the Nikon Eclipse Ti2‐E (Nikon Instruments, Melville, NY, USA) with a Plan Apo λ ×20 objective and a Nikon DS‐Ri2 color camera (final resolution 0.37 μm/pixel) in Columbus. Images were exported as TIFF files for further processing and alignment to the sequencing data. After imaging, permeabilization of the tissue was performed for 15 min. The released poly‐adenylated mRNA from the tissue sections was then captured by the barcoded primers on the Visium slide. Through reverse transcription, in the presence of template‐switching oligonucleotides, the captured mRNA was converted to spatially barcoded, full‐length cDNA. Thereafter, second strand synthesis was performed. Finally, cDNA was released from the capture areas on the slides by denaturation and subsequently used for PCR amplification. The total number of cycles ranged between 16 and 23 and was determined based on Cq values (see supplementary material, Table S7). Afterwards, enzymatic fragmentation and size selection (using solid‐phase reversible immobilization beads) was performed for optimization of the cDNA amplicon size. Finally, through end repair, A‐tailing, adaptor ligation, and PCR amplification, P5, P7, i7, and i5 sample indexes, and TruSeq Read 2 adapter sequences were added to generate a sequencing‐ready indexed library. The spatial gene expression libraries were sequenced on an Illumina NovaSeq6000 (Illumina, Inc., San Diego, CA, USA) with a target of ∽125 million paired‐end reads per sample, or 50,000 reads per spot for each sample.

### Drawing masks on top of H&E‐stained images

Masks were manually made to annotate fat, gaps, and folds in the tissue. Masks were then aligned to the sequencing data. This was done in Photoshop with the image that was used in SpaceRanger (10x Genomics, Inc., Pleasanton, CA, USA: https://www.10xgenomics.com/support/cn/software/space-ranger, accessed: 8 August 2024). After layer duplication, the wand tool or magic wand tool was used to select the region of interest on the Visium image. For each area annotated as fat, a separate mask was made. Each region of interest was selected with the ‘select and mask’ function, with transparency set at 100% and saved as a new PNG document. Masks were then loaded into the Seurat object.

### Immunofluorescent FN1 staining

To visualize fibrotic tissue in skeletal muscle biopsies, serial sections were collected immediately after the Visium section (5 μm thick) on glass slides (Epredia™ Superfrost™Plus Adhesion Microscope Slides, J1820AMNZ, Thermo Fisher Scientific, USA). During cryosectioning, the glass slides were kept in the cryostat chamber during sectioning to maintain RNA integrity. After sectioning, the slides were stored at −80 °C until further processing.

For fibrotic tissue estimation, we stained for fibronectin 1 (FN1). Glass slides were brought to RT and fixed in ice‐cold acetone for 5 min, after which they were air dried at RT for 30 min. A hydrophobic barrier was drawn using the ImmEdge™ Pen (H‐4000, Vector Labs, CA, USA). Hereafter, sections were washed once using 1× PBS and blocked for 1 h at RT using 1XPBS/0.05%Tween/5% horse serum. Subsequently, slides were incubated overnight with primary antibody for FN1 [Santa Cruz, Fibronectin (EP5), sc‐8422, Mouse monoclonal, 1:400, diluted in 1× PBS/0.05%Tween/5%FBS] at 4 °C. The next day, the slides were washed three times with 1× PBS and incubated for 60 min at RT with the Alexa Fluor 647 conjugated secondary antibody (Life Technologies, A21235, Goat‐anti‐mouse, Alexa Fluor 647, IgG, 1:500). Slides were then washed three times for 5 min with 1× PBS, mounted with DAPI (ProLongTM Gold antifade reagent with DAPI, P36935, Invitrogen, Thermo Fisher Scientific, Eugene, OR, USA) and covered with a cover slip. The slides were imaged on the ZEISS Axio Scan.Z1 slide Scanner (Carl Zeiss, Cambridge, UK) with a ×20 objective. DAPI was imaged in the 350‐nm channel and FN1 in the 674‐nm channel. The ZEN 2012 software (blue edition, Carl Zeiss) was used to process the images.

### Visium data preprocessing

Image analysis, base calling, and quality scoring of the sequencing data were performed with the Illumina data analysis pipeline RTA (version 3.4.4) (Illumina, Inc.), and the demultiplexed FASTQ data were generated using BClConvert (version 3.10.5) (Illumina, Inc.) We manually aligned the fluidic area and outlined the tissue in the histological image using Loupe Browser (version 6.4.0) (10x Genomics, Inc., https://www.10xgenomics.com/support/software/loupe‐browser/, accessed: 28 February 2023). The alignment results with the FASTQ data were mapped using the ‘count’ function in Space Ranger version 2.0.1 (10x Genomics, Inc., https://www.10xgenomics.com/support/cn/software/space‐ranger, accessed: 8 August 2024) with the GRCh38 human genome reference (refdata‐gex‐GRCh38‐2020‐A).

The data were analyzed with the Seurat package (version 5.0.3) (https://satijalab.org/seurat/, accessed: 11 October 2025) in R (version 4.3.2) (https://www.r-project.org/, accessed: 11 October 2025). For each tissue section, we first manually identified the fat, folds, and gaps based on the histological image as described above. Then we removed spots located in gaps, folds, and spots with low counts that were not identified as fat (nSpatial_Count < 30 & label ! = 'fat'). Next, we filtered out all the mitochondrial and ribosomal genes. After filtering, all the datasets were normalized using log normalization with the ‘NormalizeData’ function.

### Visium spot annotation using module scoring

To annotate the nonadipocyte spatial spots, we assigned marker genes to predefined modules (connective tissue and muscle fibers) based on the literature (supplementary material, Table S1). We then calculated the module score for each defined module based on the average expression of these marker genes using the ‘AddModuleScore’ function in Seurat. Based on the mixture distribution of the module scores for each module, we further divided the spatial spots for each sample into high‐ and low‐level module score groups, with the threshold selected based on the majority votes from iterating the ‘normalmixEM’ function from the mixtools R package (version 2.0.0) (https://cran.r-project.org/web/packages/mixtools/, accessed: 13 March 2024) 500 times.

First, spots within the high‐level connective tissue module score group were assigned to the ‘Connective Tissue’ module. Then the remaining spots were subdivided using the muscle fibers module score. Spots within the high‐level muscle fibers module score group were assigned to the ‘Muscle Fibers’ cluster, while the remaining spots were classified as ‘not assigned’.

To further subdivide spots within the ‘Muscle Fibers’ module into different muscle fiber types, we compared the log‐normalized expression levels for the myosin gene specific to the following muscle fiber types: Type I: *MYH7*; Type IIa: *MYH2*; Type IIx: *MYH1*. Each spot was further reassigned to a specific muscle fiber type based on the corresponding myosin gene with the highest expression level.

### Differential gene expression analysis

Differential gene expression analysis was performed using the pseudo‐bulk approach implemented in the muscat R package (version 1.13.1) (https://www.bioconductor.org/packages/release/bioc/html/muscat.html, accessed: 11 August 2024). We conducted comparative analysis between healthy control (HC) and DYS at both the sample level and the module level. At the sample level, pseudo‐bulk profiles were generated for each individual sample by aggregating counts across all spots. For the module‐level comparison, pseudo‐bulk profiles were created by summing counts for spots within each annotated cluster per sample for each condition. Pseudo‐bulk profiles with fewer than 10 cells were excluded from the analysis. Additionally, lowly expressed genes were filtered out using filterByExpr function with the default setting in the edgeR package (version 3.42.4) (https://bioconductor.org/packages/release/bioc/html/edgeR.html., accessed: 8 November 2024). Next, differentially expressed genes (DEGs) across conditions were identified using the edgeR method in muscat, with a selection cutoff |log_2_(fold‐change)| > 0.5 and an adjusted *p* value < 0.05 using the Benjamini–Hochberg procedure.

### Deconvolution

We used the CARD R package (version 1.1) (https://github.com/YMa-lab/CARD, accessed: 26 October 2024) to deconvolute the Visium data and infer the cell type composition for each spot. CARD is based on a nonnegative matrix factorization model, utilizing the cell type markers from a single‐nucleus RNA sequencing (snRNAseq) reference dataset for deconvoluting spatial transcriptomics data. In addition, CARD also leverages spatial information to enable accurate deconvolution in spatial transcriptomics. For the reference data, we used snRNAseq from Suárez‐Calvet *et al*, which includes human skeletal muscle from healthy and DMD individuals [10]. First, we removed all mitochondrial and ribosomal genes from the snRNAseq data. Next, we excluded the ‘B/T cells’ cell type from the reference data because there were too few cells (*n =* 188) to obtain reliable estimates for this cell population. Then we identified marker genes of the remaining nine cell types (fast fibers, slow fibers, regenerative fibers, adipocytes, faps, endothelial cells, satellite cells, smooth muscle cells, and monocytic cells) using Seurat::FindAllMarkers (min.pct = 0.1, logfc.threshold = 2.5, only.pos = TRUE) and selected the top 50 marker genes per cell type under the threshold of adjusted *p* value < 0.05 based on Bonferroni correction as the input for CARD deconvolution.

### Cell‐cell communication analysis

LIANA+ (lianapy version 0.1.8; [49]) was used to compute cell‐cell communication (CCC) scores between cell types surrounding adipose tissue. To study distance‐dependent communication events, we established three adipose‐adjacent layers manually. We specified a combination of sample of origin and layer as the ‘sample_context’ for the spots used as input to the ‘liana.method.rank_aggregate.by_sample’ function. The cell type assignments after deconvolution were used as the group option. LIANA's Consensus database was used as a ligand–receptor (L–R) resource. We applied two filtering steps to identify relevant L–R interactions. First, we excluded pairs that fell below an expression threshold of 0.6 (i.e. retaining only interactions where both ligand and receptor were expressed in ≥ 60% of spots for any given cell type pair). Second, we compared the expression levels between the adipose‐adjacent layers and the remaining muscle tissue. Only L–R pairs showing at least a two‐fold higher mean expression in the adipose‐surrounding layers were retained for further analysis.

To capture CCC trends along the layers, we used Tensor‐Cell2Cell version 0.6.8 [50]. In brief, we reduced the dimensionality of the computed communication scores using tensor factorization. The input tensor contained the CCC scores between pairs of cell types in each sample context (i.e. in which layer and sample each spot is located) and for each L–R pair. In total, 21 contexts were defined across 7 cell types and 649 L–R pairs, which resulted in a tensor with dimensions 21 × 649 × 7 × 7. After factorization, we obtained 15 factors of CCC trends across each of the original dimensions in four different matrices. For the factorization, the ‘cell2cell.analysis.run_tensor_cell2cell_pipeline’ function was used with robust optimization, random initialization, and NumPy's Singular Value Decomposition (SVD) solver.

### Spatial fat analysis

We applied SPATA2 (version 3.0.0) (https://themilolab.github.io/SPATA2/, accessed: 30 October 2024) to identify genes whose expression patterns are influenced by fat tissue. For samples containing fat areas, we imported the space ranger output to SPATA2 object using the command SPATA2::initiateSpataObjectVisium(). Next, we annotated the fat areas based on the H&E‐stained images by manually outlining their boundaries using SPATA2::createImageAnnotations(). We then applied the function ‘SPATA2::spatialAnnotationScreening()’ to screen for genes with nonrandom patterns along the distance from the annotated fat areas to all tissue spots. Only spatially variable genes with nonzero counts were included in the analysis based on the function ‘SPATA2:: removeGenesZeroCounts’ followed by ‘SPATA2:: getSparkxGenes(…, threshold_pval = 0.05)’. Pattern genes associated with the fat trajectory were selected based on an adjusted *p* value < 0.05 (Benjamini–Hochberg correction) and a root mean squared error (RMSE) < 0.25, fitting with the descending or ascending models predefined by SPATA2.

### Statistical analyses

To assess significant differences in module annotation percentages and cell type percentages following deconvolution, independent two‐sample *t*‐tests were conducted in R (version 4.3.2). This method tests the transformed proportions of modules and cell types across samples between HC and DYS cohorts. Prior to statistical testing, percentage data were subjected to an arcsine square root transformation to stabilize variance and approximate normality.

## Results

### A dystrophinopathy spatial transcriptomics dataset

In this study, we generated a spatial transcriptomics dataset from skeletal muscle biopsies to investigate dystrophinopathies. This included a total of *n =* 12 samples (average age; standard deviation), which included *n =* 8 DYS samples (7.9; 2.8) and *n =* 4 sex‐matched controls (23.5; 6.4) (Table 1). Biopsies were obtained from the lower and upper leg muscles at two sites (Nationwide Children's Hospital, Columbus, OH, USA, and Leiden University Medical Center, Leiden, the Netherlands). The spatial transcriptomics datasets were then generated using Visium from 10x Genomics.

**Table 1:** Patient characteristics of included samples in this study.

Cohort	Sample	Age (years)	Muscle	Sex	Mutation nomenclature	Mutation
HC	1	26	Gastrocnemius	M		
	2	14	Hamstring	M		
	3	26	Hamstring	M		
	4	28	Gastrocnemius	M		
DYS	1	11	Quadriceps (U)	M	c.(264+1_265‐1)_(357+1_358‐1)del	Del exon 5
	2	8	Quadriceps (VL)	M	c,4294C>T; p.(Gln1432*)	c.4294C>T (exon 31)
	3	12	Quadriceps (VL)	M	c.(649+1_650‐1)_(1602+1_1603‐1)dup	Dup exon 8–13
	4	8	Quadriceps (VL)	M	c.(357+1_358‐1)_(5922+1_5923‐1)del	Del exon 6–41
	5	8	Tibialis anterior	M	c.(6438+1_6439‐1)_(6762+1_6763‐1)del	Del exon 45–46
	6	6	Quadriceps (VL)	M	c.(93+1_94‐1)_(6290+1_6291‐1)dup	Dup exon 3–43
	7	7	Quadriceps (U)	M	c.4600C>T; p.Gln(1534*)	c.4600C>T (exon 33)
	8	3	Quadriceps (VL)	M	c.(264+1_265‐1)_(357+1_358‐1)del	Del exon 5

U, unknown which muscle was biopsied; VL, vastus lateralis.

**Figure 1:**
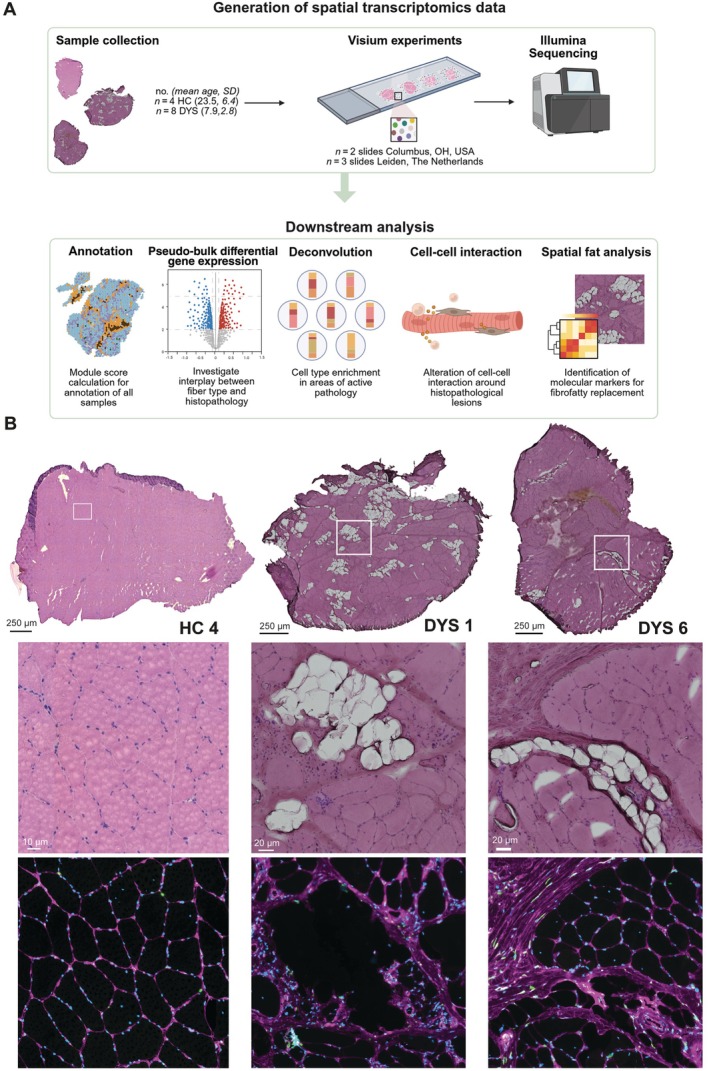
Study overview and representative histological features of included samples. (A) Schematic overview of study and (B) representative H&E images of samples for each group (HC, DYS) as well as a *FN1* staining for fibrosis. Created with BioRender.com.

Using histological and transcriptomic data we annotated and compared sections across groups. To further elucidate the cell type contribution to histopathological lesions in the tissue, we utilized a deconvolution strategy using a previously published snRNAseq dataset [10] and assessed cell–cell interactions in these areas of active pathology. Lastly, we identified genes showing a spatial expression with a gradient surrounding intramuscular fat (Figure 1A).

Muscle biopsies of HCs were histologically more homogeneous in tissue composition, whereas DYS samples showed a variety of histopathological lesions, as reflected by the H&E staining and confirmed by immunofluorescent staining of fibrosis and connective tissue marker fibronectin (FN1) on consecutive sections (Figure 1B; supplementary material, Figures S1–S3). Biopsies presented fibrosis, fat, inflammation, centralized nuclei, and variation in fiber size. The presence and extent of these histopathological lesions varied across samples.

Upon processing the samples, a total of 1,125,766,335 paired‐end reads covering a total of 19,437 spots (8,183 spots HC; 11,254 spots DYS) were obtained with a mean of 60,324 reads per spot (Table 2).

**Table 2:** Overview of Next Generation Illumina sequencing results used in this study.

Cohort	No.	No. reads Columbus	No. reads Leiden	Total reads	No. spots under tissue	Mean reads per spot	Median UMI counts per spot	Median genes per spot
HC	1	‐	179,385,012	**179,385,012**	1,596	1,12,397	896	368
	2	69,722,279	‐	**69,722,279**	1,286	54,216	147	93
	3	‐	72,827,267	**72,827,267**	1,911	38,110	1,658	505
	4	‐	163,077,843	**163,077,843**	3,390	48,106	1,181	463
DYS	1	57,881,174	15,777,089	**73,658,263**	1,646	44,750	2,944	1,050
	2	34,898,373	22,415,086	**57,313,459**	805	71,197	4,566	937
	3	28,357,053	‐	**28,357,053**	1,136	24,962	3,853	587
	4	70,365,987	64,408,453	**134,774,431**	1,718	78,448	2,758	866
	5	‐	82,090,390	**82,090,390**	856	95,900	904	432
	6	60,427,341	11,199,628	**71,626,969**	1,596	44,879	5,422	1,236
	7	66,184,467	13,392,381	**79,576,848**	1,398	56,922	422	232
	8	70,840,919	42,515,602	**113,356,521**	2,099	54,005	5,463	1,321

UMI, Unique Molecular Identifier.

### Spatial transcriptomic data highlight a loss of type IIx fibers in dystrophinopathies and increased connective tissue

**Figure 2:**
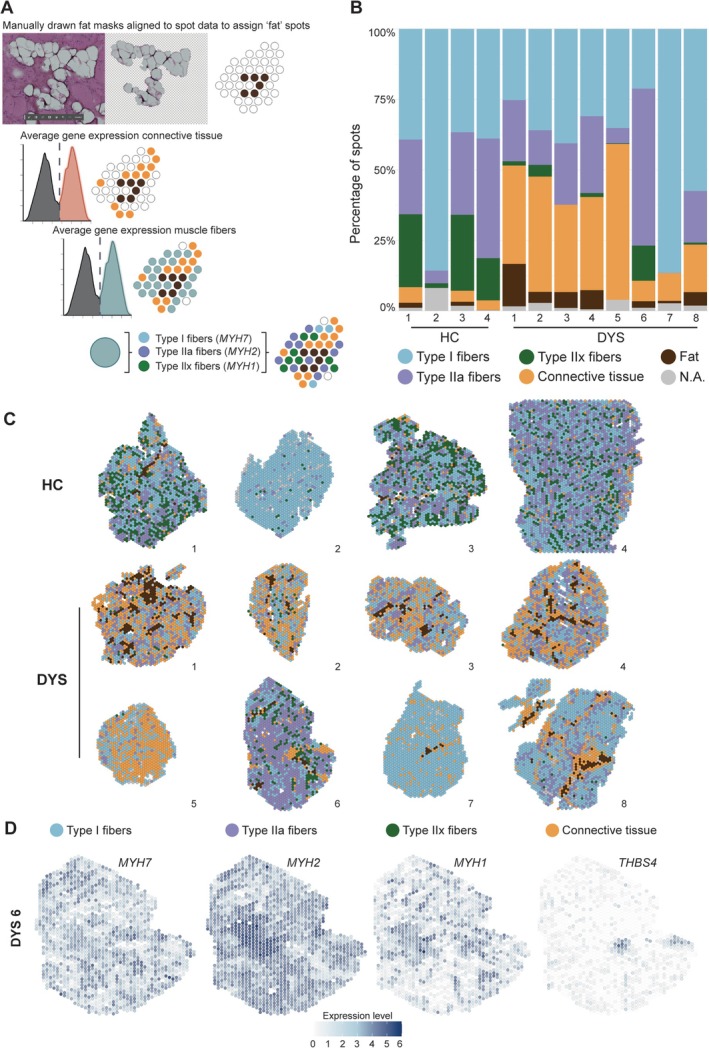
Annotation based on gene expression with module scored samples enables comparison across groups and samples. (A) Assignment of modules to spots based on presence of fat masks, expression of connective tissue markers, and expression of muscle markers. (B) Stacked barplot (0–100%) representing the composition of modules across all samples. (C) Spatial representation of module scores across samples. (D) Representative expression of specific marker genes per module in DYS sample 6. NA, not assigned.

To annotate Visium spots in each skeletal muscle sample, we employed a multistep approach combining histological examination and expression of marker genes (Figure 2A; Materials and methods). First, fat spots were manually assigned based on assessment of the H&E stained tissue images. Next, we used module scores to identify connective tissue spots based on marker genes associated with the extracellular matrix, such as *COL1A1*, *COL1A2*, and *THBS4* (supplementary material, Table S1). Similarly, we identified muscle fibers based on the module score and subsequently subdivided them into primary fiber types based on corresponding marker gene expression (Type I fibers: *MYH7*; Type IIa fibers: *MYH2*; Type IIx fibers: *MYH1*). Figure 2D presents how the annotation of connective tissue and different fiber types align with the marker gene expression pattern in sample DYS 6.

Annotation results showed that the tissue composition differed greatly across samples (supplementary material, Table S2). As expected, myonuclei represent the predominant module in our samples (Figure 2B,C; Table 3), with Type I fibers as the largest submodule among the myonuclei, followed by Type IIa and Type IIx.

**Table 3:** Average percentage of modules across HC and DYS samples.

		Type I	Type IIa	Type IIx	Connective tissue	Fat	NA
**Average %**	**HC**	50.12	25.68	17.33	3.24	0.80	2.83
	**DYS**	42.66	20.76	2.49*	26.55*	**4.90**	2.65

Percentages marked in bold show modules for which large differences were observed across HC and DYS samples, while * marks *p* values <0.05.

NA, not assigned.

Comparing the different groups, we found that Type IIx were significantly more frequent in HC samples (17.33%) compared to DYS samples (2.49%) and that areas marked as connective tissue were more prevalent in DYS samples (26.55%) compared to the HC samples (3.24%) (independent two‐sample *t*‐test; *p < 0.05). Areas annotated as fat were also increased in DYS samples (4.90%) compared to HC samples (0.80%), although this difference was formally not significant (independent two‐sample *t*‐test; *p* = 0.06). A general reduction in muscle fibers was observed for both Type I fibers (50.12% in HC and 42.66% in DYS) and Type IIa fibers (25.68% in HC and 20.76% in DYS).

### Differential gene expression analysis linked to fiber type composition

**Figure 3:**
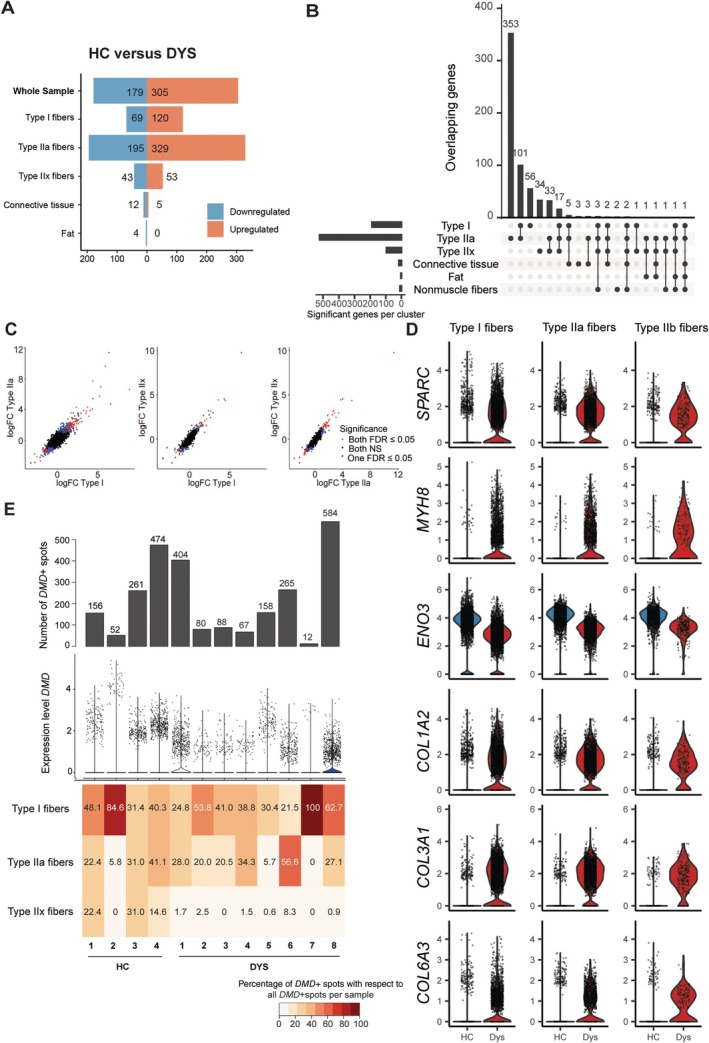
Differential gene expression analysis highlights differences between HC and DYS. (A) Number of DEGs (up‐ and downregulated) from pseudo‐bulk analysis across modules. (B) UpsetR plot showing the overlap in DEGs between HC and DYS across modules. (C) Scatterplot showing correlation in logFC across fiber types. Black dots represent genes that are not differentially expressed, blue dots are genes that are differentially expressed in only one of the two fiber types (false discovery rate < 0.05), while red dots represent genes that are differentially expressed in both fiber types. (D) Example of DEGs in HC (blue) and DYS (red) samples. (E) The absolute number of *DMD*+ spots in the samples, followed by the expression level of *DMD* in these samples and the percentage of *DMD*+ spots per assigned fiber type with regard to all *DMD*+ spots per sample.

To compare the transcriptomes, we performed pseudo‐bulk differential gene expression between the two groups (HC versus DYS) for each annotated region. The comparison showed that most DEGs were found in modules annotated as Type I and IIa fibers (Figure 3A), most likely due to the low number of Type IIx fibers in the DYS group. A total of 56 genes were differentially expressed exclusively in Type I fibers, 353 were expressed solely in Type IIa fibers, and 34 were specific to Type IIx fibers (Figure 3B). Although this analysis suggests that DEGs are mostly cell type specific, the log fold‐changes showed good correlation across fiber types, suggesting that the dystrophic signature was shared across fiber types (Figure 3C). DEGs shared across fiber types included an increased expression in regeneration markers such as *SPARC* and fetal myosin (*MYH8*), an increased collagen expression (*COL1A2*, *COL3A1, COL6A3*), and reduced expression of genes involved in muscle contraction and metabolism (*MYBPC2*, *ATP2A1*, *ANO3*, *PGM1*) (Figure 3D). Some of these genes (such as *MYH8*, *COL3A1*, *PGM1*, *ENO3*) showed a congruent directional increase in connective tissue, most likely due to neighboring muscle fibers contributing to the signature. Interestingly, genes previously reported as serum biomarkers in dystrophinopathies such as *CA3* [11] and *ART3* [12] were found to be differentially expressed in muscle fibers and connective tissue, respectively. All DEGs are reported in the supplementary material, Table S3.

In dystrophinopathies, active pathology predominantly occurs at the myofiber level where dystrophin is primarily expressed. *DMD* gene expression was variable in both the number of positive spots and the expression level (Figure 3E). *DMD* expression levels were generally higher in healthy individuals compared to DYS subjects. Considering the different fiber types, we observe that *DMD* expression tended to be higher in regions enriched in slow twitch (Type I) fibers compared to fast twitch ones (Type IIa/IIx), especially in the diseased samples (Figure 3E, supplementary material, Figure S4 for spatial plots). Higher dystrophin expression was found in DYS sample 5, which was, however, affected by the low number of muscle cells (especially type IIa and IIx) and was also the only biopsy obtained from the tibialis anterior muscle.

### Enrichment of FAPs and adipocytes in histological lesions revealed by deconvolution

**Figure 4:**
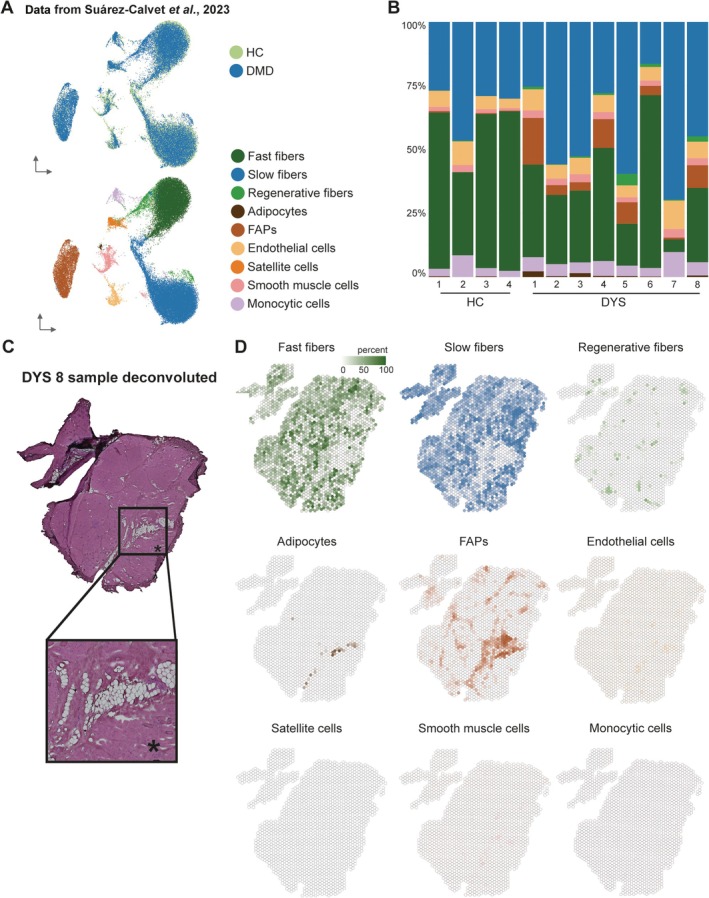
Deconvolution of samples shows cell type composition across groups and its association with histopathological alterations. (A) SnRNAseq reference dataset used for deconvolution with CARD derived from HC and DMD nuclei of different myo and non‐myo nuclei. (B) Stacked barplot of cell type composition for all samples included in dataset. (C) H&E‐stained sample DYS 8 with zoomed‐in area showing intramuscular fat and fibrosis. (D) Deconvoluted cell types per spot ranging from 0% to 100%.

The spots used to capture gene expression had a diameter of 55 μm, and the center‐to‐center distance was 100 μm. This resulted in areas of the tissue for which cDNA was not synthesized (as they were not covered by a spot) and spots that captured more than one cell. The number of cells captured by each individual spot varied depending on the tissue context, with some spots capturing a single muscle fiber and others capturing multiple cells. To determine the cell type contribution in each Visium spot, we used deconvolution based on a reference snRNAseq dataset, in which both healthy controls and DMD samples were sequenced (Figure 4A; [10]). This reference dataset contained myonuclei originating from slow, fast, and regenerating fibers, as well as from smooth muscle cells. Moreover, nonmyonuclei were also present such as adipocytes, FAPs, endothelial cells, satellite cells, and monocytic cells (Figure 4A).

The most prevalent cell types contributing to the transcriptomic profiles of the Visium spots were myonuclei, primarily slow and fast fibers, with large variation across samples (Figure 4B). Dystrophic samples showed a lower percentage of fast fibers (31.74%) compared to HC samples (54.15%). The reduction in fast twitch fibers was evident in both deconvolution and module score analyses, suggesting an increased fragility of these fibers in patients with dystrophinopathy compared to slow twitch fibers. FAP presence was higher in DYS samples (7.27%) compared to the HC (average 0.30%) along with adipocytes (0.71% in DYS compared to 0.14% in HC and regenerating fibers 1.37% in DYS and 0.14% in HC) (Figure 4B; Table 4). Smooth muscle cells were also found to be elevated in DYS samples.

**Table 4:** Average percentage of deconvoluted cell types across HC and DYS samples.

	Fast fibers	Slow fibers	Regenerative fibers	Adipocytes	FAPs	Endothelial cells	Satellite cells	Smooth muscle cells	Monocytic cells
**HC**	54.15	33.12	0.14	0.14	0.30	6.10	0.01	1.73	4.31
**DYS**	31.74*	44.01	1.37*	**0.71**	7.27*	6.82	0.03	2.69*	5.37

Percentages marked in bold show modules for which large differences were observed across HC and DYS samples, while * marks *p* values <0.05 in single‐tailed *t*‐test.

Cell type percentages were variable within groups, and especially in DYS patients. As an example, some samples showed sizable increases in FAPs (DYS 1: 18.29% and DYS 4: 11.33%), while other biopsies, characterized by less ongoing damage, showed lower FAP estimates in the deconvolution results (e.g. FAPs in DYS 2: 3.84% and DYS 7: 0.72%). An overview of the percentages of all deconvoluted cell types by sample can be found in the supplementary material, Table S4.

Importantly, spatial data allow visualization of the deconvolution results on histological sections, enabling us to assess the presence of cell types at areas of histological lesions. For example, in DYS 8, adipocytes, identified through deconvolution, were mostly present in areas where fat was visible by histology, while FAPs were present around fat patches and in areas of endomysial fibrosis (Figure 4C,D). The detail of the H&E image in Figure 4C shows how the most severely affected region in the sample was characterized by substantial fibrofatty replacement and how this region was enriched with FAPs and adipocytes, suggesting that FAPs are particularly present in areas of active disease, while adipocytes are located in areas of end stage pathology (Figure 4D).

### CCC around intramuscular fat depositions reveal crosstalk between FAPs, adipocytes, and myofibers

**Figure 5:**
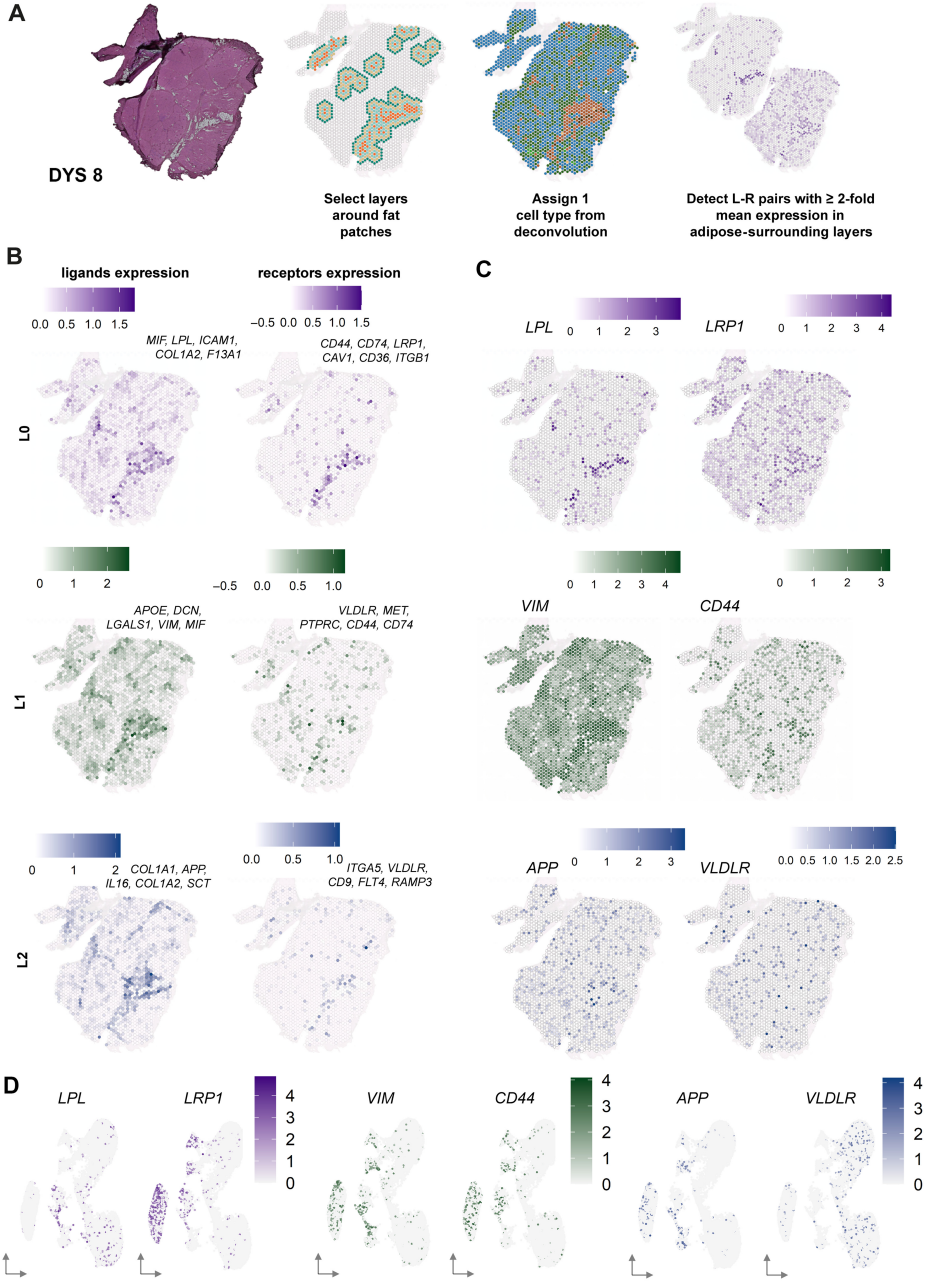
Cell–cell communication across layers surrounding intramuscular fat infiltration. (A) DYS8 sample is shown to present the analysis. Panels present H&E staining, schematic of layer selection, and cell type annotation prior to detection of ligand–receptor (L–R) pairs across layers. (B) Combined expression scores of the top five ligands and their corresponding receptors across different layers (L0, L1, and L2). (C) Spatial expression patterns of one representative L–R pair per layer. (D) Expression of the highlighted L–R pairs plotted in snRNAseq data indicating potential sender and receiver cell types.

To examine which cells are actively contributing to the histopathological lesions leading to the appearance of fat patches in dystrophic skeletal muscle, we performed CCC analysis around areas of intramuscular fat depositions. This analysis allows for the detection of L–R pairs enriched in layers surrounding fatty infiltrations (supplementary material, Table S5). Areas annotated as fat based on the H&E images were considered as layer 0 (L0). Spots in close proximity were annotated by expanding radially outward: immediate neighboring spots were assigned to layer 1 (L1), followed by layer 2 (L2) and layer 3 (L3). All remaining spots were left unassigned. Based on the previously described deconvolution results, every spot of the tissue was also assigned one cell type (the cell type with the highest percentage presence per spot based on the deconvolution results). Thereafter, we detected CCC across the different layers (L0–L1; L1–L2 and L2–L3) based on the expression of L–R pairs in at least 60% of the spots with at least two‐fold higher expression in these layers compared to the unassigned spots located far from fat patches. Sample DYS 8 was used to visualize L–R pairs (Figure 5), while the other samples were used for further visualization (supplementary material, Figures S5–S7).

L–R pairs (L^R) enriched between L0 and L1 (meaning closest to the fat) were as follows: *MIF^CD44/CD74*, *LPL^LRP1*, *ICAM1^CAV1*, *COL1A2^CD36*, and *F13A1^ITGB1*. A combined, average expression score shows that the expression of these ligands and receptors was indeed localized in the areas surrounding the fat patches (Figure 5B‐L0). In the *LPL^LRP1* pair, *LPL* was specifically expressed in the adipocytes (senders), and *LRP1* was expressed in the neighboring layer enriched in FAPs (receivers) bordering the fat patch (Figure 5C‐L0). Confirmation of sender and receiver cell types was obtained from the snRNAseq data, where adipocytes were shown to express *LPL*, and FAPs showed expression of *LRP1* (Figure 5D). L–R pairs (L^R) that were enriched in the communication between L1 and L2 cells were as follows: *APOE^VLDLR*, *DCN^MET*, *LGALS1^PTPRC*, *VIM^CD44*, *MIF^CD44/CD74*. Spatial plots of the average expression of these ligands and receptors highlight areas surrounding the fat patch but also reflect areas where fibrotic tissue deposition was observed (based on H&E images) and that are enriched in FAPs (deconvolution results; Figure 4D) (Figure 5B‐L1). The senders in particular seemed to colocalize with areas enriched in FAPs. Spatial plots of the *VIM^CD44* L–R pair showed that the pair is specific for areas surrounding histological lesions, as *VIM* expression was visible throughout the section, with the highest expression surrounding histological lesions, where the *CD44* receptor was present to capture the ligand (Figure 5C‐L1). Expression in the snRNAseq dataset suggests the senders to be the FAPs and endothelial cells and receivers to be FAPs, satellite cells, and smooth muscle cells (Figure 5D). Lastly, L–R pairs (L^R) that were detected more distant from the fat between L2 and L3 were as follows: *COL1A1^ITGA5*, *APP^VLDLR*, *IL16^CD9*, *COL1A2^FLT4*, *SCT^RAMP3*. Here, the average expression of the L–R pairs was outside of the fat patches and more in the fibrotic and surrounding areas (Figure 5B‐L2). The *APP^VLDLR* pair (Figure 5C‐L2) shows that *APP* was expressed by a combination of FAPs, satellite cells, and endothelial cells, whereas *VLDLR* was expressed mostly by muscle fibers (slow and fast) (Figure 5D). As FAPs were central in all L–R pairs, we reclustered the snRNAseq data to identify whether specific FAPs subtypes were enriched in proximity to tissue lesions. FAP subcluster 19 (supplementary material, Figure S8A,B) was particularly enriched in these L–R pairs. These FAPs expressed marker genes such as *COL3A1*, *CFD*, *GSN*, *APOD*, *C3*, and *CXCL14*, which were previously connected to both adipogenic and fibrogenic commitment (supplementary material, Figure S8C).

### Characterizing spatially relevant adipose tissue markers

Substitution of muscle with adipose tissue is considered a hallmark of dystrophinopathies and the end histological stage of the pathological process. Importantly, muscle fat fraction is considered to be a prognostic biomarker for patients suffering from a dystrophinopathy. To identify marker genes of adipose tissue in dystrophinopathies, we assessed what genes were enriched in areas of muscle substituted by fat and what genes showed a gradient toward fat patches.

**Figure 6:**
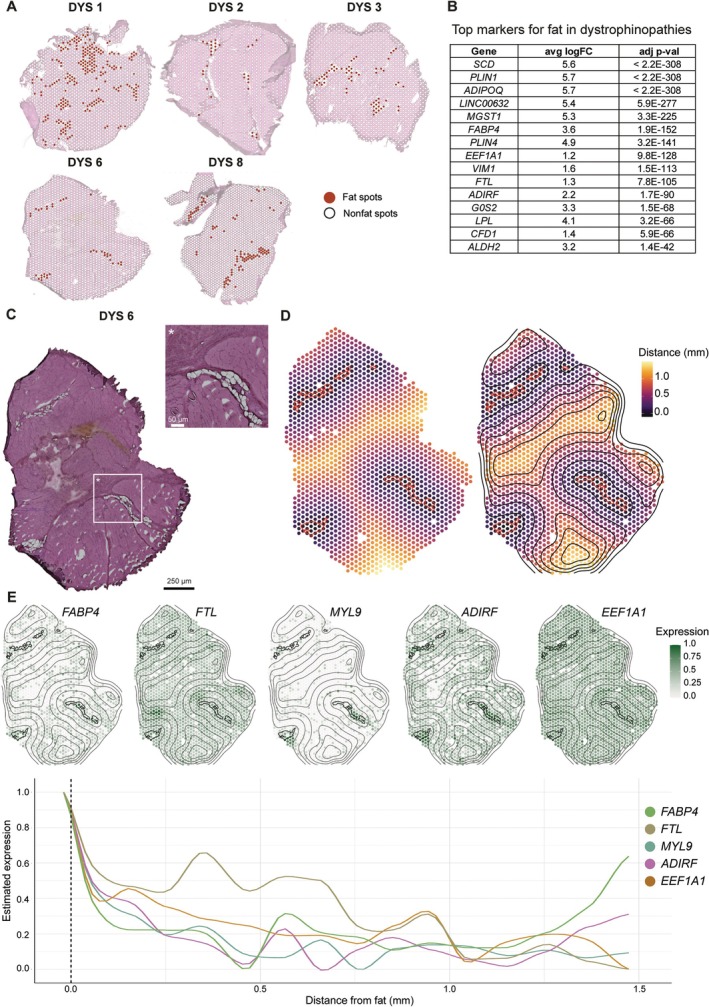
Identification of spatially relevant adipose tissue markers. (A) Selection of ‘fat’ spots (red) in DYS tissues for differential gene expression analysis compared with ‘nonfat’ spots (white) within the grouped samples. (B) Top overlapping genes in fat spots in DYS samples ranked by adjusted *p* value (based on Log2FC). (C) H&E‐stained image of DYS sample 6 with a zoomed‐in region surrounding fibrofatty infiltration was used to allow visual comparison between histological observations and gene expression. (D) Left: spots are colored according to their distance from the fat masks (red line). Right: lines indicating gradient direction and orientation based on distance from the fat masks. (E) Spatial and line plots of expression patterns of representative genes that have a decreasing expression pattern with increasing distance from the fat areas.

We selected five DYS samples with substantial fat infiltration and compared gene expression in areas annotated as with all ‘nonfat’ areas (Figure 6A). The top 15 marker genes (based on Log2FC) associated with fat areas are listed in Figure 6B (supplementary material, Figure S9 for spatial expression patterns). Known adipocytes markers such as *ADIPOQ* and a lipogenic enzyme *SCD* were identified, in line with previous reports [10, 13, 14]. Most detected genes are well described and linked to lipid metabolism and adipogenesis, e.g. *PLIN1*, *PLIN4*, *SCD*, *LPL*, and *FABP4*, but have not been associated with dystrophinopathies before [15, 16, 17, 18, 19].

To identify genes with informative spatial patterning, especially surrounding borders of fatty infiltrations, we employed spatial fat analysis using SPATA2 [20]. This analysis takes the distance from the defined fat areas into account and aims to identify genes that may play an active role in the transition from muscle to fat, in areas where the muscle has not yet been replaced by adipose tissue. For this analysis, the same samples, i.e. those with substantial fat infiltration, were used (Figure 6A). Fat masks were drawn on top of the H&E image for every sample (Figure 6C highlights DYS 6 as an example). Thereafter, the distances from the area of interest were calculated, and lines were added to visualize the distance based on the proximity to the fat masks (Figure 6D). SPATA2 identified genes that align with predefined expression patterns such as linear descending, early descending, instantly descending, gradual peak, medium peak, and small peak. Most identified genes fell in the descending instant, gradual, or linear category (supplementary material, Table S6). Genes that were identified in multiple samples were *FABP4*, *FTL*, *MYL9*, *ADIRF*, and *EEF1A1* (Figure 6E; supplementary material, Figure S10), suggesting that these genes could be used to identify areas of muscle that are committed to transition toward fat but not yet visible in histology.

## Discussion

Dystrophinopathies, mostly known as Duchenne and Becker muscular dystrophy, are characterized by tissue damage and clinical decline due to suboptimal levels of dystrophin. In this study, we used spatial transcriptomics to identify gene expression signatures in relation to histological lesions in skeletal muscle biopsies of patients affected by dystrophinopathy. We mapped molecular changes to tissue alterations while retaining the spatial context, which is crucial for a better understanding of the histopathology. The obtained data show how fast twitch fibers are particularly sensitive to reduced dystrophin levels and how FAPs are the drivers of tissue lesions leading to fibrotic and adipose tissue deposition. Spatial analysis allowed us to model patterns in tissue and interaction across cell types present in proximity to these lesions, leading to a model of how such histological alterations come to exist.

Previous gene expression studies have enabled the identification of genes and pathways affected in dystrophinopathies using microarrays and bulk RNA‐seq [21]. Such analyses, which are affected by the cell type composition and gene expression changes, are often interpreted as a change in tissue composition. Common examples are the increase in *COL1A1* and *COL1A2* expression as a surrogate of fibrotic tissue deposition. Our data show that a common dystrophic gene expression signature is present in areas marked by muscle fibers, independently of the fiber type. While the number of spots mapped to Type I and IIA fibers made it possible to identify a large set of DEGs; differential gene expression analysis was affected in Type IIX fibers as they were severely reduced in DYS samples, supporting earlier findings that fast twitch fibers are preferentially affected in dystrophinopathies [22]. The gene expression profile in dystrophic muscle fibers encompassed regeneration markers, myogenic markers, extracellular matrix genes, genes related to fibrosis, inflammation, and metabolic processes [23, 24]. A unifying pattern across DYS biopsies was the deposition of fibrotic and adipose tissue. Deconvolution analysis showed that FAPs were enriched in areas of fibrosis and surrounding adipocytes, suggesting that active/ongoing pathology converges on the presence of FAPs, while the end‐stage pathology is represented by the presence of adipocytes. While FAPs are key to successful muscle regeneration in healthy conditions, we observed these cells as the main drivers of histological lesions. Importantly, the deconvolution strategy provides an estimate of cell types present at each location; in our case the analysis was performed using previously published snRNAseq data obtained from independent individuals in the largest snRNAseq study available at the time of analysis. Our data are consistent with recent spatial transcriptomics studies showing that FAPs aggregate in fibrotic regions of DMD patients, as revealed by MERFISH mapping [25]. Similarly, PDFGA^+^ FAPs accumulate in fibrotic and regenerating areas in D2‐*mdx* mice [26], supporting their spatial association with sites of tissue damage and remodeling. A recent omics study supports FAPs involvement with different expression patterns, but it lacks spatial context [27]. To understand how fibrotic and adipose tissues are deposited in muscle, we studied the co‐expression of L–R pairs and the patterning of gene expression in contained areas. The goal of this analysis was to determine similarities and differences between L–R pairs leading to fibrotic and adipose lesions by relying on their proximity to fat patches. CCC and expression in proximity to fat would provide information on the commitment of FAPs to the adipogenic lineage, while the same analyses farther away from fat patches would make it possible to study FAPs committed to the fibrogenic lineage. In our study, several L–R pairs were enriched in proximity to fat patches (L0–L1), some of which are known to be involved in lipid metabolism and adipogenesis. Among these, the LPL^LRP1 pair stood out due to its strong representation in the analysis and compelling biological rationale. Lipoprotein lipase (LPL) is a key enzyme in lipid metabolism, hydrolyzing triglycerides present on chylomicrons and VLDL into free fatty acids, which can then be used or stored as energy by the tissue. This reaction typically occurs in blood vessels, consistent with our snRNAseq data showing LPL expression in smooth muscle cells and endothelial cells, in addition to adipocytes. LRP1, which binds LPL, has been implicated in adipocyte differentiation and lipid uptake [28, 29]. We show that LRP1 is predominantly expressed in a subset of FAPs that was notably enriched in L–R pairs (see combination of Figure 5D and supplementary material, Figure S8A). Moreover, in fibroblasts and smooth muscle cells, *LRP1* controls the trafficking of platelet‐derived growth factor receptor β (*PDGFRβ*), which in turn is a marker linked to FAPs and pre‐adipocytes [30]. Disrupted expression of *LRP1* on smooth muscle cells has been shown to result in elevated levels of *PDGFRβ* expression and atherosclerotic lesions and fibrosis [31, 32]. Experiments aimed at interfering with LPL^LRP1 interactions could shed light on the net contribution of such interactions to adipocyte differentiation. Farther away from the fat patches, we identified interactions between *VIM* and *CD44*, which are known to mediate a highly conserved crosstalk across immune cells such as dendritic cells (DCs) and T cells. It is thought that these interactions promote the antigen presentation and activation of autoreactive T cells [33, 34]. Finally, on the layer farthest from the fat, we identified the *VLDLR* receptor. This receptor is present in the brain, adipose tissue, and vascular endothelial cells, as well as heart and skeletal muscle [35, 36]. The interaction between *APP* and *VLDLR* has mostly been studied in Alzheimer's disease, a neurodegenerative disorder, and seems to be essential for normal brain functioning, neuronal development, and lipid metabolism [35, 37]. Its role in neuromuscular disorders remains to be uncovered.

This study has a few limitations. Given that fewer muscle biopsies are now being performed for diagnostic purposes, we could only retrospectively access samples that were collected at different clinical sites and from different muscle groups. Moreover, the obtained healthy controls were not completely healthy due to anterior cruciate ligament surgery and were significantly older. Despite this limitation, the identification of common genes across muscles further supports the strength of the associations identified in this work. The heterogeneity of the biopsies in the DYS group was large, with two biopsies showing less prominent tissue lesions compared to the other six samples: one biopsy was rather small and showed no adipose tissue deposition, and a second biopsy was enriched in slow twitch fibers, which are less prone to damage, and showed less pronounced histopathology. Despite these limitations, we could identify a shared dystrophic signature across fiber types. Importantly, this comparison allowed us to propose genes that relate to dystrophin dosage/expression and could be used to monitor the effects of dystrophin reintroduction by dystrophin restoring therapies. Finally, the resolution of the Visium data did not allow us to study the spatial expression of single cells, and we used a deconvolution strategy to estimate cell types at spatial locations. Future work should aim to bridge this gap and use spatial transcriptomic technologies that allow to study individual cells in histological sections. Nevertheless, the transcriptome‐wide coverage allowed for the identification of novel markers (e.g. *LPL*, *LRP1*, *VLDLR*, *APP*, *PLIN1*, *FTL*) that would normally not be included in targeted panels due to their previously unknown link to dystrophinopathies.

To conclude, while multiple snRNAseq and spatial gene expression studies exist in preclinical models [27, 38, 39, 40, 41, 42, 43, 44, 45, 46, 47], this is one of the first spatial transcriptomic studies on human skeletal muscles. The analysis enabled the identification of marker genes of histological lesions such as adipose tissue deposition and to propose genes and cell types involved in the transition of muscle to fat. Higher‐resolution studies using high‐resolution spatial technologies will make it possible to clarify the net effect of cell types in an active pathology niche.

## Author contributions statement

LGMH performed the wet lab experiments and parts of the analysis and drafted the first version of the manuscript. QM performed parts of the analysis and wrote parts of the manuscript. SN and KMF provided biopsies of a healthy control and seven DYS samples and allowed for collaborative execution of the wet lab experiments by LGMH in their lab. CNR performed the CCC analysis and wrote the methods on this part. Under the supervision of LGMH, JvdW performed the immunofluorescent staining on the skeletal muscle. JK contributed to the implementation of the SPATA2 pipeline for the skeletal muscle tissue. EHN and HEK provided biopsies of healthy controls and one DMD sample from Leiden University Medical Center and The Hague. JD‐M and RGN provided the snRNAseq dataset. PS, AM, MvP and AA‐R supervised the work, led by PS and AM. All authors provided feedback and comments on the manuscript draft.

## Supporting information


**Figure S1.** H&E images of samples included in the study
**Figure S2**. FN1 stained HC samples
**Figure S3**. FN1 stained DYS samples
**Figure S4**. Spatial expression of the *DMD* gene
**Figure S5**. CCC DYS layers and cell types
**Figure S6**. L–R pairs detected across layers and plotted across samples (DYS 1 and DYS 2)
**Figure S7**. L–R pairs detected across layers and plotted across samples (DYS 3 and DYS 6)
**Figure S8**. Marker subset of interesting FAPs based on CCC analysis
**Figure S9**. Adipogenic marker genes across samples
**Figure S10**. SPATA2 plot genes across samples
**Table S1**. Genes used for module scored annotation
**Table S2**. Module annotation numbers per sample for all modules
**Table S3**. Differential gene expression across diseases and modules
**Table S4**. Deconvolution numbers per sample for all cell types
**Table S5**. CCC results with 0.6 resolution
**Table S6**. Genes with informative spatial patterning detected with SPATA2
**Table S7**. Technical details of Visium Spatial Gene Expression slide processing

## Data Availability

Spatial transcriptomics count data are available at https://cellxgene.cziscience.com/collections/f4774e03‐b5a7‐473b‐99d6‐eeb80592c5db. All original code has been deposited on GitHub https://github.com/Qirongmao97/NMDhuman_spatial and is publicly available as of the date of publication. The used snRNAseq data are available upon reasonable request through the corresponding author of that dataset [10].
