# Supplementary material for: Unraveling the spatial landscape of dystrophinopathies: a transcriptomic approach to Becker and Duchenne muscular dystrophies
Source: J Pathol. 2026 May 1;269(3):363–80. doi: 10.1002/path.70067 (PMC13238356; doi:10.1002/path.70067)
Supplement: Supplementary file 1 — Figure S1. H&E images of samples included in the study Figure S2. FN1 stained HC samples Figure S3. FN1 stained DYS samples Figure S4. Spatial expression of the DMD gene Figure S5. CCC DYS layers and cell types Figure S6. L–R pairs detected across layers and plotted across samples (DYS 1 and DYS 2) Figure S7. L–R pairs detected across layers and plotted across samples (DYS 3 and DYS 6) Figure S8. Marker subset of interesting FAPs based on CCC analysis Figure S9. Adipogenic marker genes across samples Figure S10. SPATA2 plot genes across samples Table S1. Genes used for module scored annotation Table S2. Module annotation numbers per sample for all modules Table S3. Differential gene expression across diseases and modules Table S4. Deconvolution numbers per sample for all cell types Table S5. CCC results with 0.6 resolution Table S6. Genes with informative spatial patterning detected with SPATA2 Table S7. Technical details of Visium Spatial Gene Expression slide processing [file PATH-269-363-s001.zip › path70067-sup-0001-FiguresS1-S10TablesS1-S7/path70067-sup-0001-FiguresS1-S10TablesS1S7.docx]

**Unraveling the spatial landscape of dystrophinopathies: a transcriptomic approach to Becker and Duchenne muscular dystrophies**

LGM Heezen, Q Mao, *et al. J Pathol* <https://doi.org/10.1002/path.70067>

**Supplementary Figures S1–S10**

**Supplementary Tables S1 and S7**

**Supplementary Tables S2–S6 are provided in separate Excel files**

**
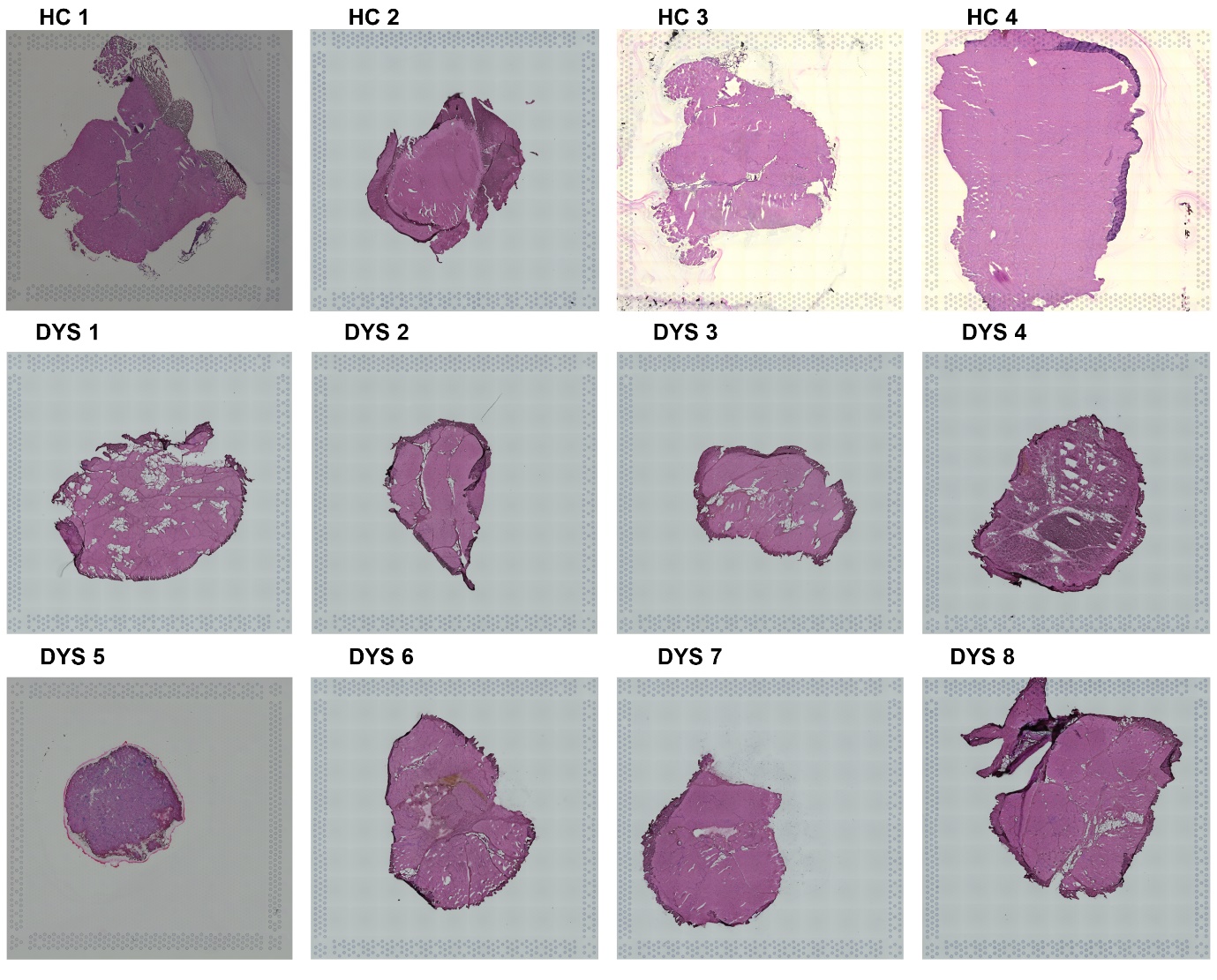
**

**Figure S1. H&E images of samples included in the study.**


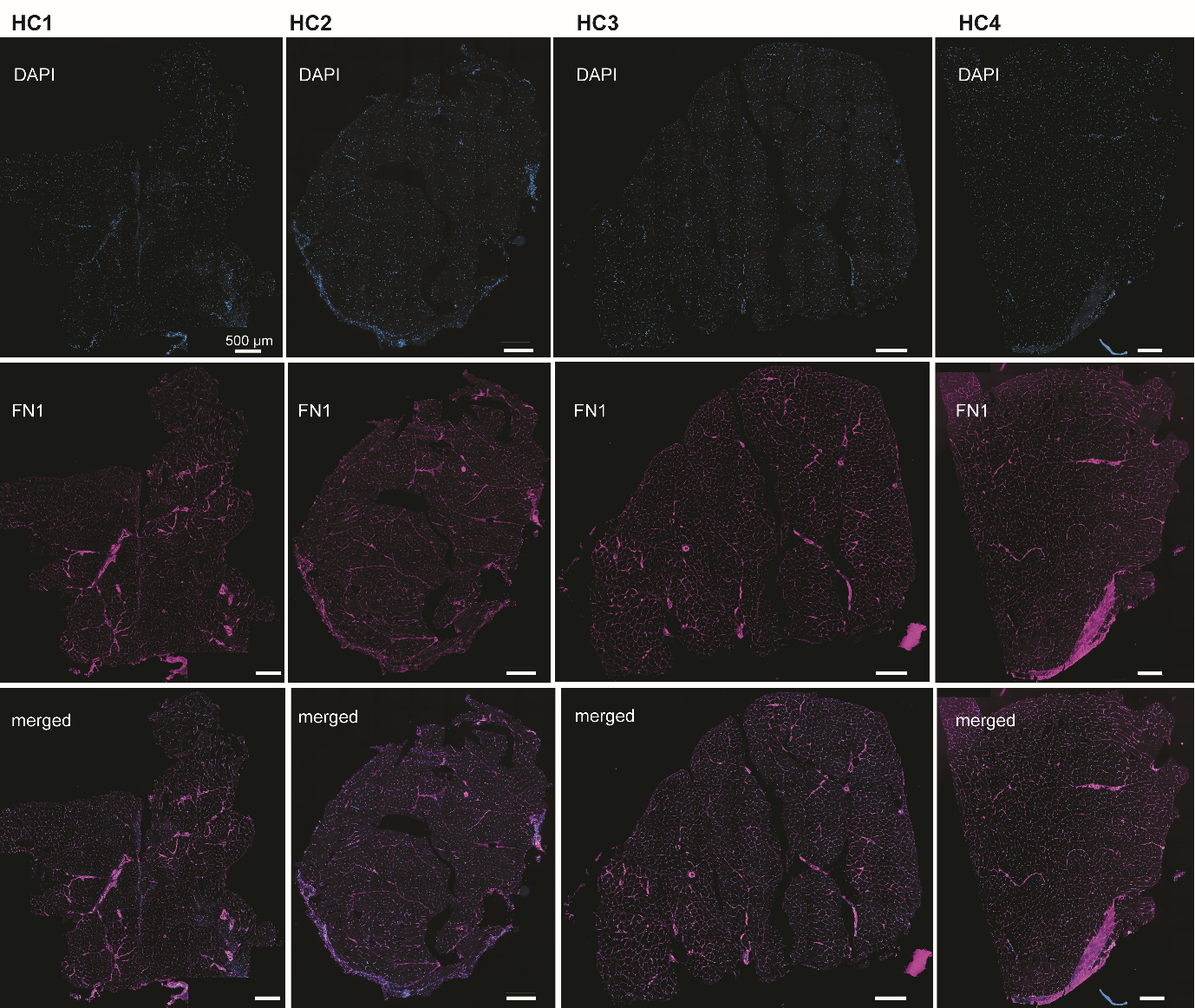


**Figure S2. FN1 stained HC samples.** All HCs stained for FN1 as a marker of fibrosis and connective tissue co-stained with DAPI highlighting the nuclei.

**
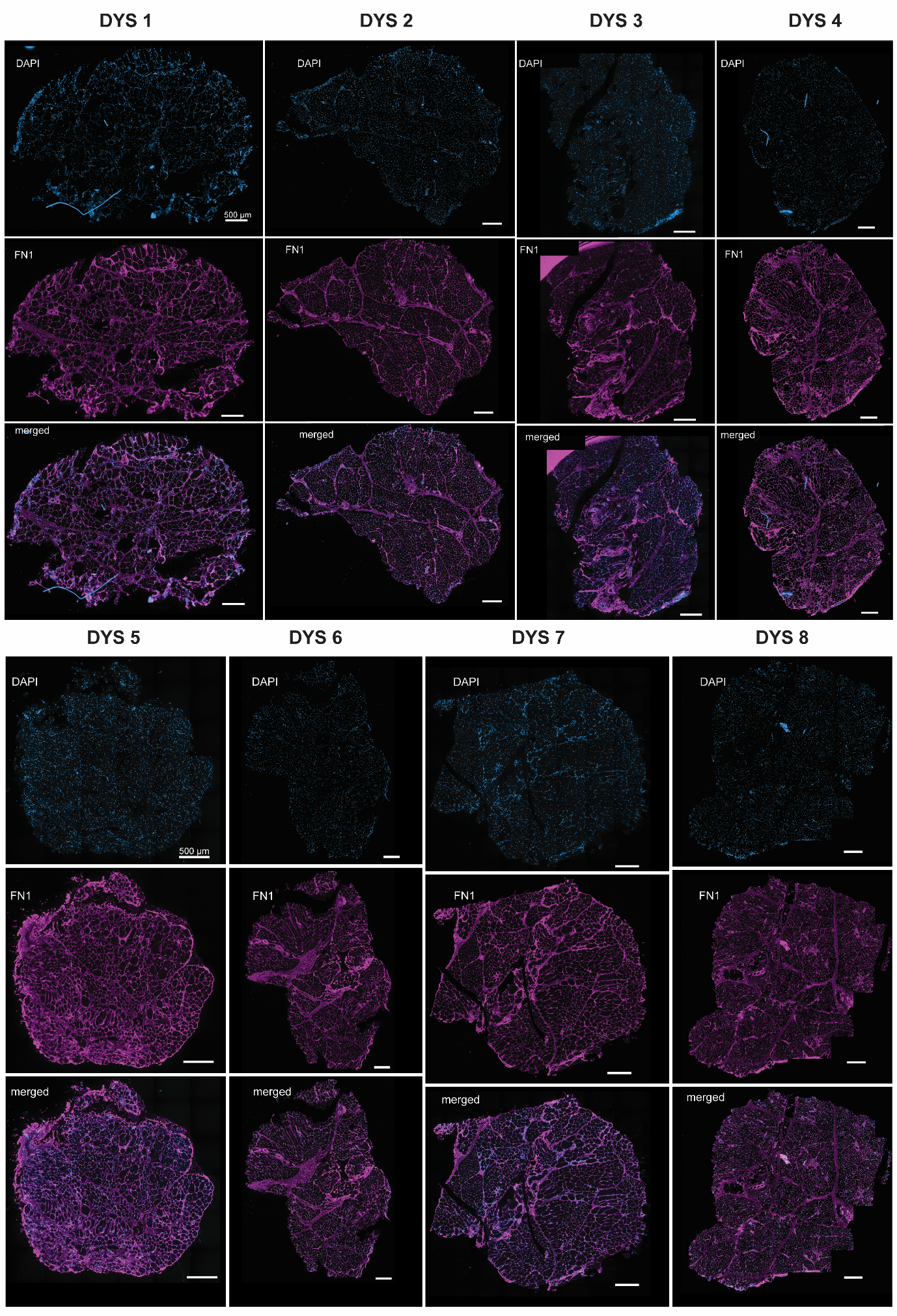
**

**Figure S3. FN1 stained DYS samples.** All DYS samples stained for FN1 as marker of fibrosis and connective tissue co-stained with DAPI highlighting the nuclei.

**
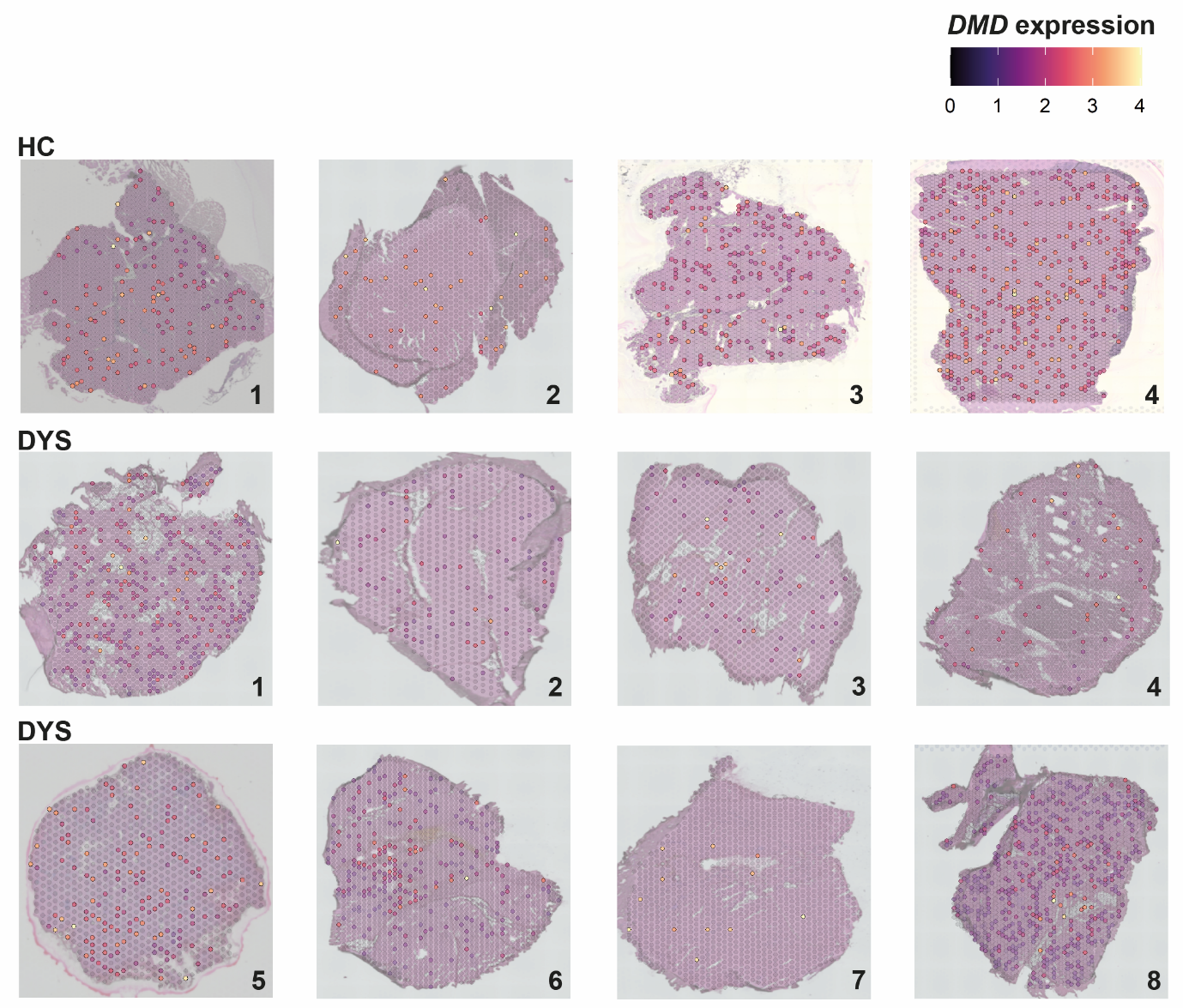
**

**Figure S4. Spatial expression of *DMD* gene.** *DMD* gene expression spatially plotted across all samples included in the study.


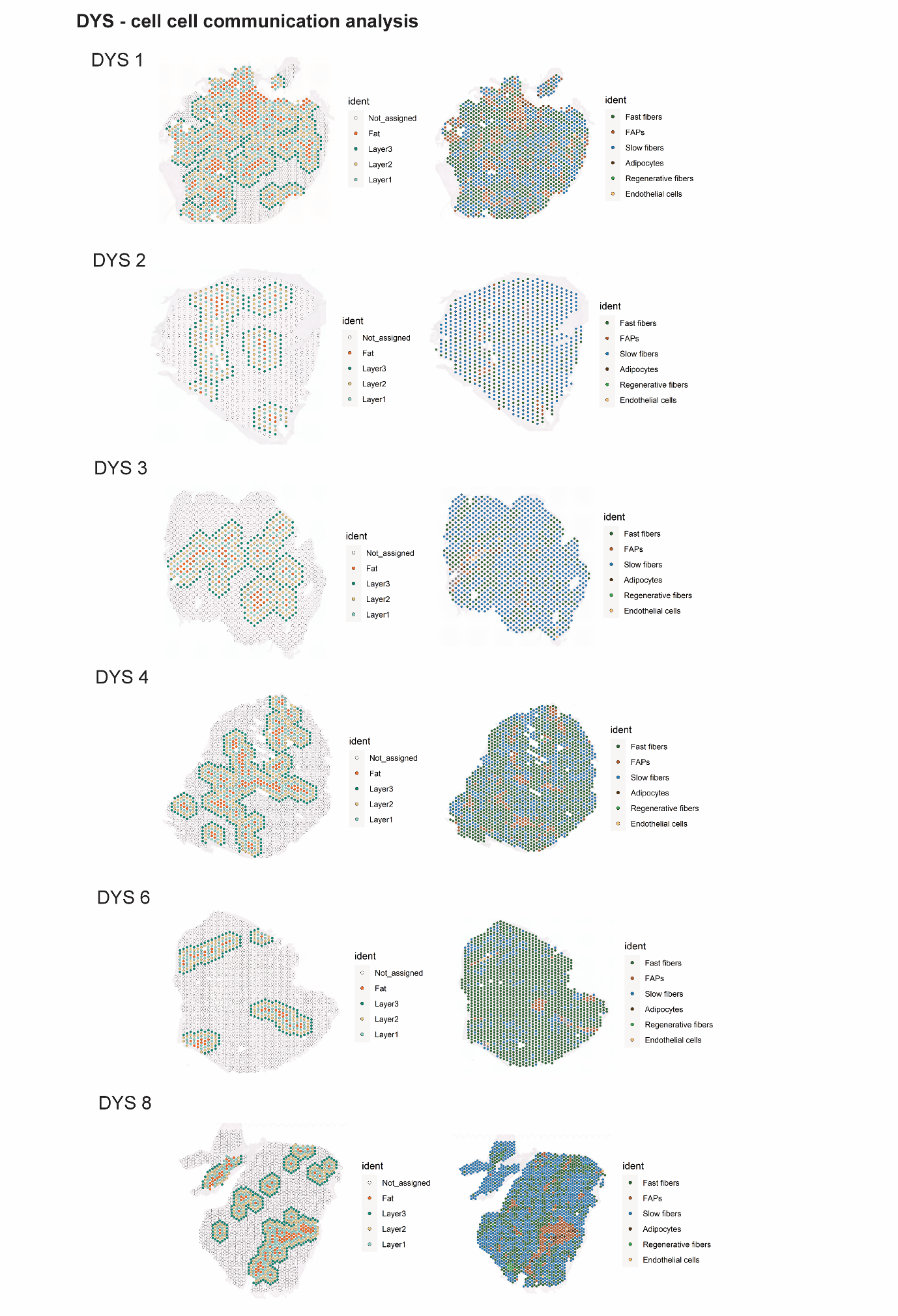


**Figure S5. CCC DYS layers and cell types.** DYS samples included in CCC analysis, fat and surrounding layers annotated included in the analysis as well as the assigned cell type.

**
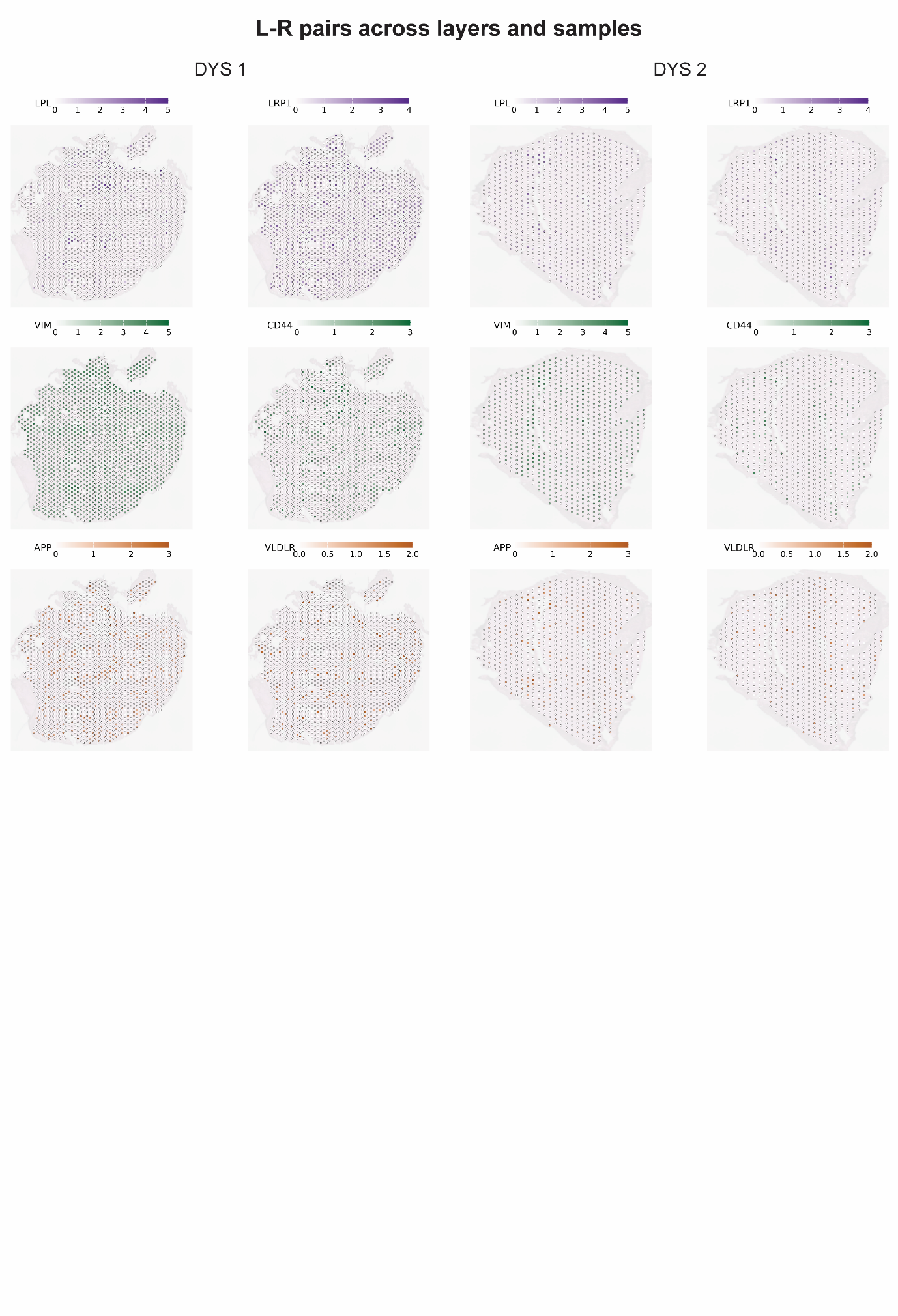
**

**Figure S6.** **L–R pairs detected across layers and plotted across samples (DYS 1 and DYS 2).**

**
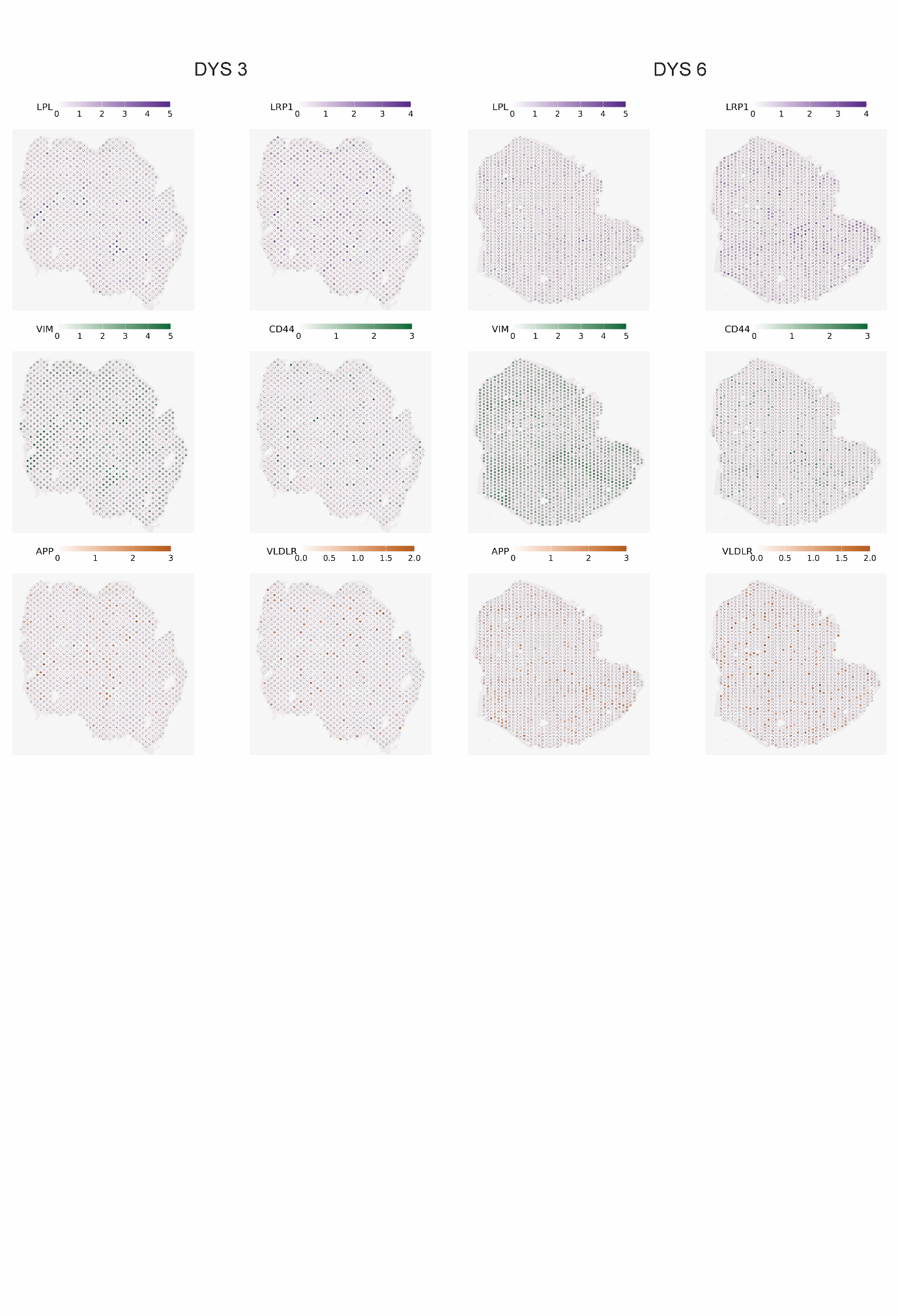
**

**Figure S7. L–R pairs detected across layers and plotted across samples (DYS 3 and DYS 6).**

*
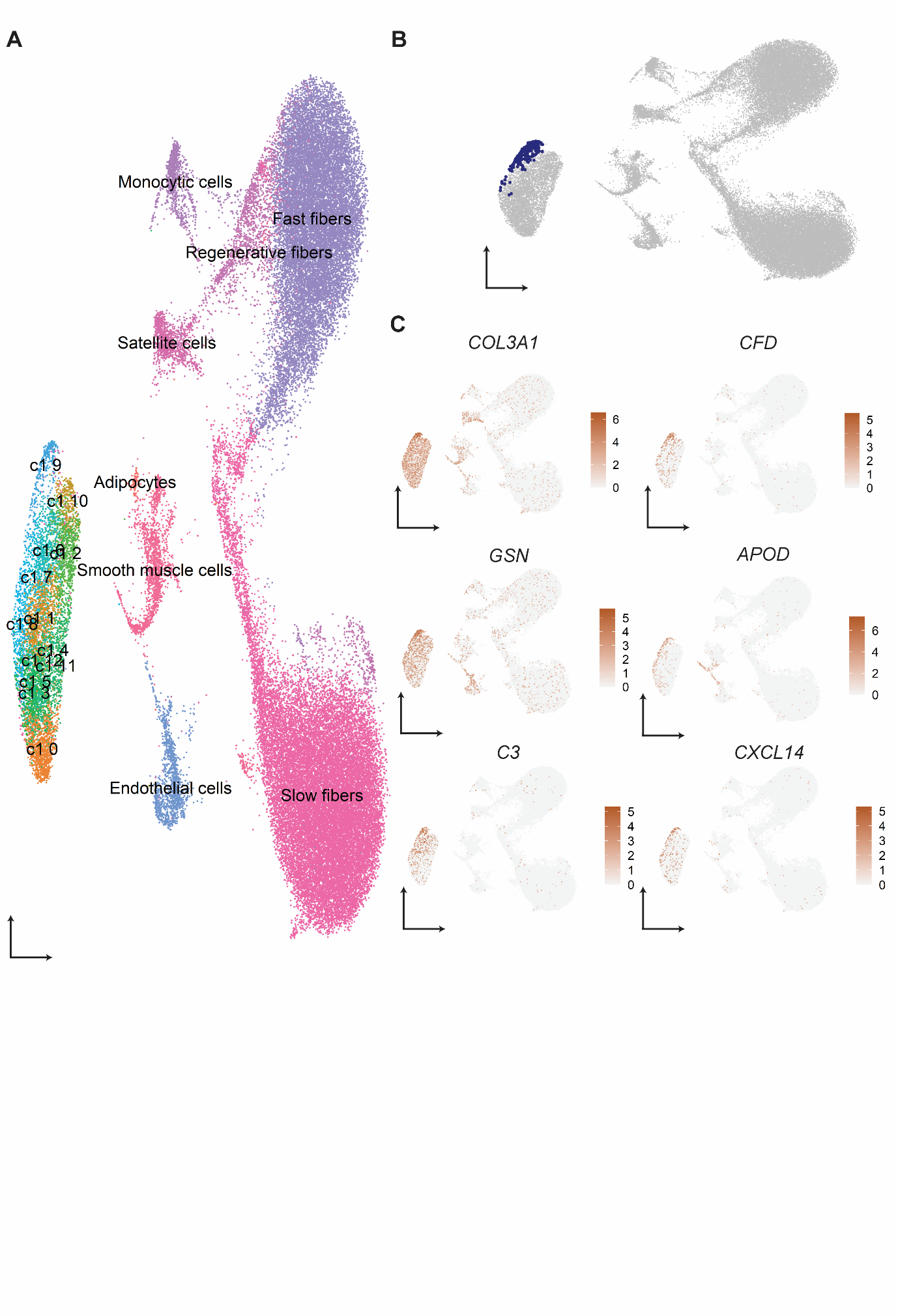
*

**Figure S8. Marker subset of interesting FAPs based on CCC analysis.** Reclustering FAPs from single-nucleus dataset to detect markers of specific FAP cluster thought to be representative of FAPs in our CCC analysis. (A) Reclustering of FAP cohort from single-cell dataset divides FAPs into multiple clusters. (B) Cluster c19 is the main cluster expressing L–R markers from our CCC analysis. (C) A few top expressing markers for this specific cluster are *COL3A1*, *CFD*, *GSN*, *APOD*, *C3*, and *CXCL14*.

**
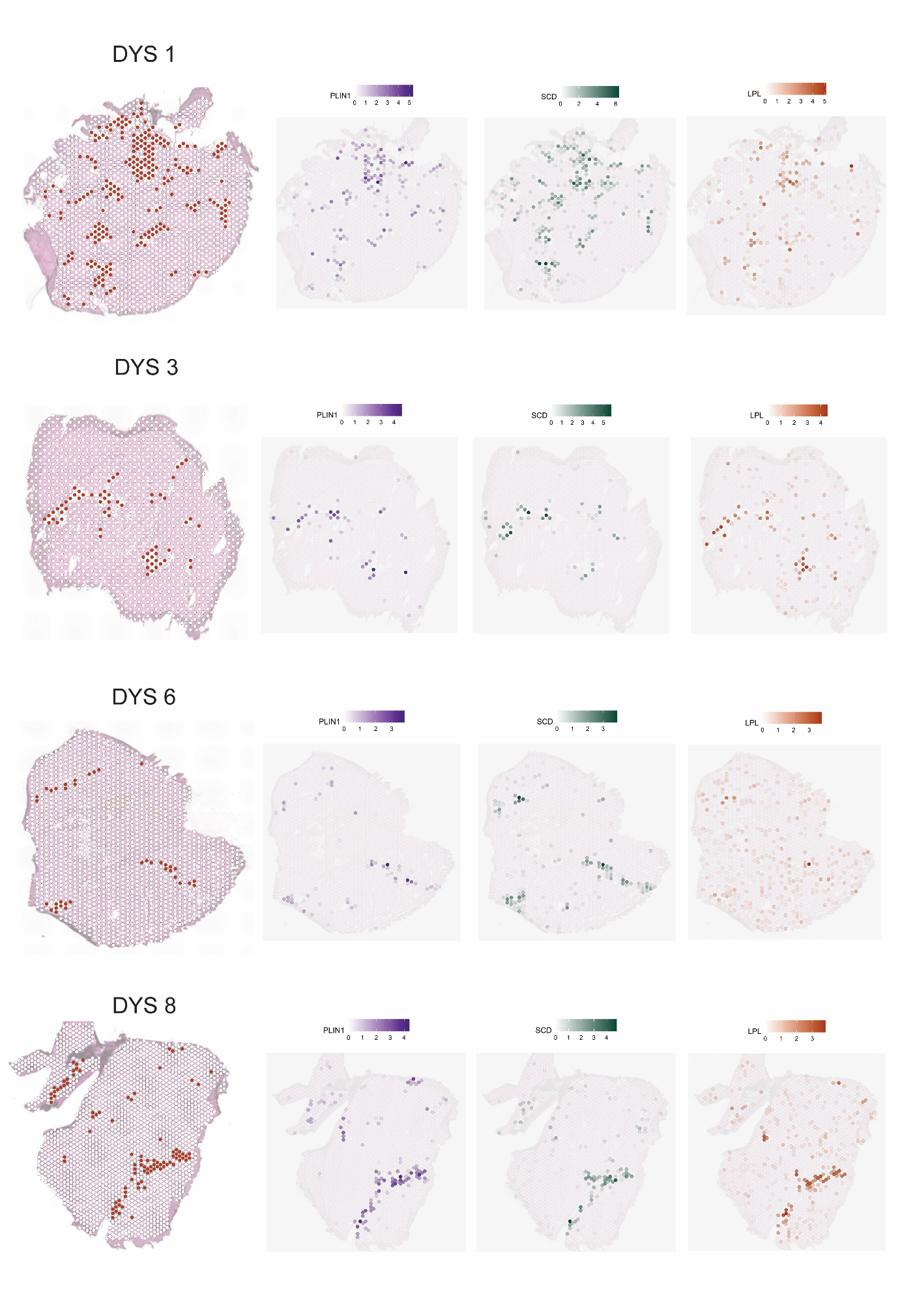
**

**Figure S9. Adipogenic marker genes across samples.** Spatial mapping of a few adipogenic marker genes (*PLIN1*, *SCD*, *LPL*) across samples included in spatial fat analysis.

**
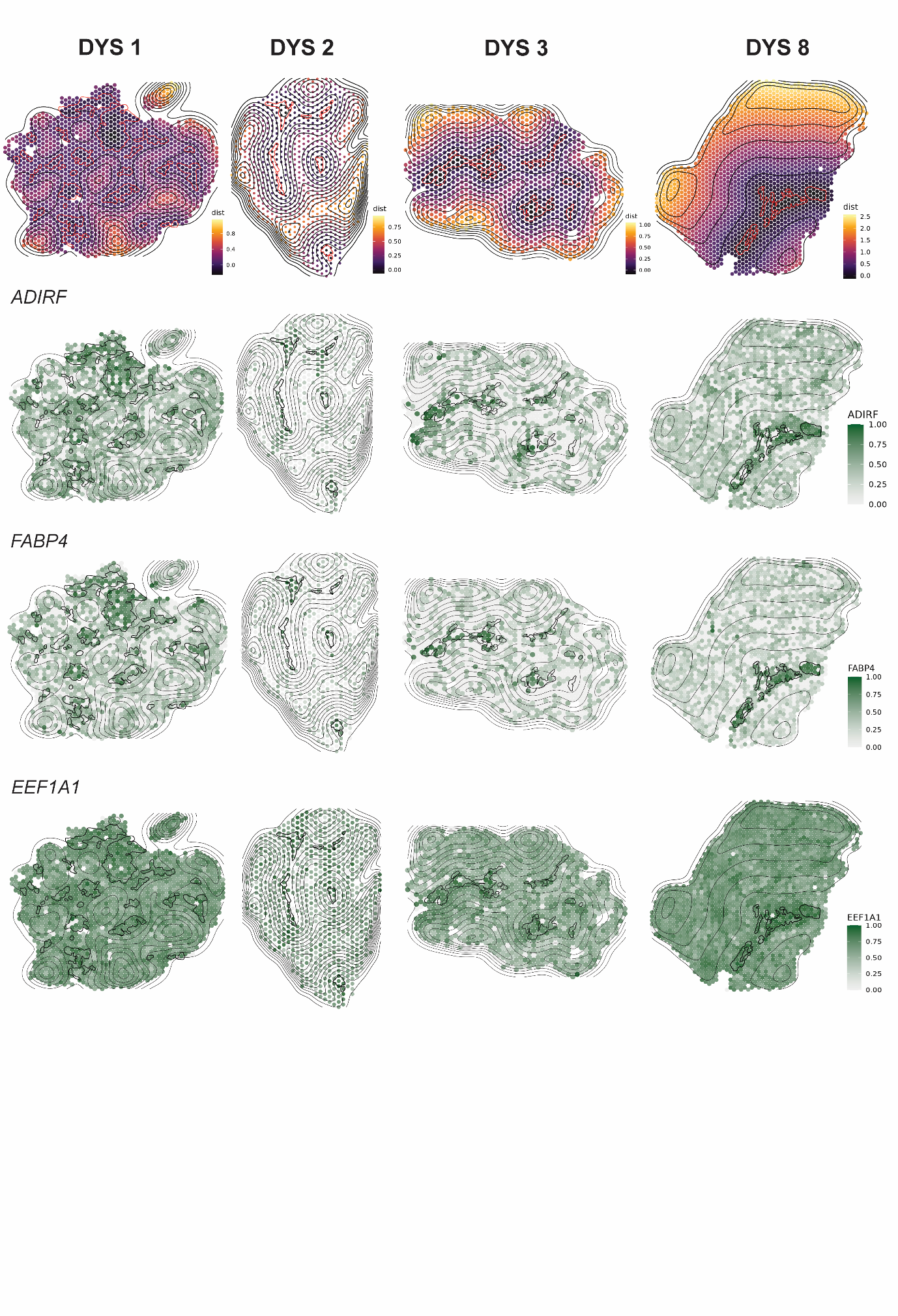
**

**Figure S10. SPATA2 plot genes across samples.** A selection of three SPATA genes (*ADIRF*, *FABP4*, *EEF1A1*) plotted spatially across samples.

**Table S1.** Genes used for module scored annotation.

| **Connective tissue** | **Muscle Fibers** | **Type I** | **Type IIa** | **Type IIx** |
| --- | --- | --- | --- | --- |
| *COL1A1* | *CKM* | *MYH7* | *MYH2* | *MYH1* |
| *COL1A2* | *TNNT3* |  |  |  |
| *THBS4* | *TPM1* |  |  |  |
| *COL3A1* | *TPM2* |  |  |  |
| *FN1* | *TNNT1* |  |  |  |
| *COL6A6* | *MYH1* |  |  |  |
| *DCN* | *MYH2* |  |  |  |
| *GSN* | *MYH7* |  |  |  |
| *BGN* | *ATP2A1* |  |  |  |
|  | *TNNI2* |  |  |  |
|  | *ENO3* |  |  |  |
|  | *PFKM* |  |  |  |
|  | *PKM* |  |  |  |
|  | *TNNI1* |  |  |  |
|  | *MYH7B* |  |  |  |

**Table S7.** Technical details of Visium Spatial Gene Expression slide processing. Technical details of Visium slide number, position of capture area, and amount of PCR cycles used in Visium Spatial Gene Expression experiments.

| **Cohort** | **Sample** | **Slide number** | **Capture area position** | **Percentage tissue coverage** | **cDNA cycle amplification** | **SI PCR cycle number** |
| --- | --- | --- | --- | --- | --- | --- |
| HC | 1 | V19N11-029 | A | 40 | 16 | 18 |
|  | 2 | V12Y31-101 | C | 25 | 19 | 18 |
|  | 3 | V11D13-059 | D | 40 | 23 | 14 |
|  | 4 | V11D13-059 | B | 67 | 20 | 13 |
| DYS | 1 | V12Y31-125 | B | 35 | 17 | 15 |
|  | 2 | V12Y31-125 | D | 19 | 17 | 16 |
|  | 3 | V12Y31-125 | A | 23 | 16 | 16 |
|  | 4 | V12Y31-101 | B | 36 | 17 | 16 |
|  | 5 | V19N11-029 | B | 18 | 16 | 18 |
|  | 6 | V12Y31-125 | C | 33 | 16 | 16 |
|  | 7 | V12Y31-101 | D | 28 | 19 | 18 |
|  | 8 | V12Y31-101 | A | 43 | 16 | 15 |
